# Evaluation of marginal and internal adaptation of implant-supported PEEK crowns fabricated by 3D printing, milling, and pressing: a micro-CT analysis

**DOI:** 10.1186/s12903-026-08150-8

**Published:** 2026-04-09

**Authors:** Aliaa Ibrahim Mahrous, Mostafa El-Shazly, Mahitab Mansour, Alshaimaa Ahmed Shabaan, Mohamed Mokhtar, Ahmed Tawfik, Mohamed Mostafa Radwan

**Affiliations:** 1 Fixed Prosthodontics Department, Faculty of Dentistry, Fayoum University, Fayoum, Egypt; 2 Prosthodontic Department, Faculty of Dentistry, October University for Modern Sciences and Arts, Giza, Egypt; 3 Dental Biomaterials, Faculty of Dentistry, October University for Modern Sciences and Arts, Giza, Egypt; 4 Oral and Maxillofacial Surgery, Faculty of Dentistry, Fayoum University, Fayoum, Egypt; 5 Research fellow at EPSRC Future Advanced Metrology Hub, University of Huddersfield, Huddersfield, UK; 6 Fixed prosthodontic Department, Faculty of Dentistry, Bani Suif University, Beni-Suef, Egypt

**Keywords:** Polyetheretherketone, Dental Implants, Additive Manufacturing, Digital dentistry, Accuracy

## Abstract

**Objectives:**

This study aimed to evaluate and compare the marginal adaptation and internal gap of implant-supported crowns fabricated from polyetheretherketone (PEEK) using CAD-CAM milling, heat pressing, and 3D printing, employing non-destructive micro-computed tomography (µCT).

**Methods:**

This in-vitro study used thirty PEEK crowns (*n* = 10 per group) that were fabricated using CAD-CAM milling (MP), heat pressing (PP), and 3D printing (3DP) and seated on standardized zirconia abutments. Marginal and internal gaps were quantitatively assessed using high-resolution µCT scanning (voxel size: 9.2 μm) at 12 predetermined locations per crown in sagittal and coronal planes. Measurements included marginal gaps (mesial and distal), finish line gaps, and internal gaps at axial walls, occlusal surfaces, and internal line angles. Non-parametric statistical tests (Kruskal-Wallis and Dunn’s post hoc with FDR correction) were applied, with significance set at *P* < 0.05.

**Results:**

All measurement sites showed statistically significant differences between the groups (*P* < 0.001), with large effect sizes. Milled crowns exhibited the smallest occlusal gaps and superior adaptation at the occlusal surface (*P* < 0.001), while 3D-printed crowns demonstrated the best adaptation along axial walls and internal angles. Pressed crowns consistently showed the largest marginal and internal gaps across most regions. All fabrication techniques demonstrated marginal gap values within the clinically acceptable threshold (< 120 μm); however, statistically significant differences were observed in both marginal and internal adaptation among the three groups (*P* < 0.001).

**Conclusions:**

Fabrication technique significantly affects the marginal adaptation and internal gap of implant-supported PEEK crowns. While milled crowns showed optimal occlusal adaptation, 3D-printed crowns provided the best internal conformity along axial surfaces. Pressed PEEK restorations exhibited the poorest adaptation. These findings underscore the importance of technique selection in optimizing clinical outcomes of PEEK-based implant prostheses.

**Clinical significance:**

Marginal adaptation and internal fit affect the biological and mechanical success of implant crowns. Milling and 3D printing showed better adaptation than heat pressing, supporting techniques that improve longevity and reduce complications.

**Supplementary Information:**

The online version contains supplementary material available at 10.1186/s12903-026-08150-8.

## Introduction

In the rapidly evolving field of restorative and implant dentistry, achieving accurate marginal and internal adaptation of prostheses remains a critical determinant of clinical success. Discrepancies at the crown-abutment interface can result in cement dissolution, microleakage, plaque accumulation, peri-implantitis, and mechanical failure [1, 2]. This is particularly important in implant-supported crowns, where the absence of a periodontal ligament limits shock absorption, making a passive and precise adaptation essential to avoid complications such as screw loosening and bone resorption [3].

A marginal gap of ≤ 120 μm is generally accepted as the clinical threshold [4], yet modern fabrication techniques aim to achieve even tighter tolerances. Traditional evaluation methods such as sectioning and replica techniques, are either invasive or limited in accuracy. In contrast, µCT offers a non-destructive, high-resolution, 3D approaches to quantitatively assess marginal and internal adaptation with excellent fidelity [5–7].

Among emerging restorative materials, polyetheretherketone (PEEK) has gained significant interest as an alternative to conventional metal and ceramic systems. Originally used in orthopedic and spinal implants, PEEK exhibits a favorable combination of mechanical resilience, biocompatibility, low plaque affinity, and a bone-like elastic modulus (~ 3–4 GPa) [8, 9]. These properties reduce stress transmission to underlying implants and enhance long-term performance.

In dentistry, PEEK is considered a promising candidate for implant-supported prostheses due to its shock-absorbing capacity, fatigue resistance, and potential for esthetic customization through veneering or surface treatment [10]. However, as a thermoplastic polymer, its dimensional stability and accuracy can vary significantly based on the fabrication method used [11].

PEEK restorations can be fabricated using three primary techniques: heat pressing, computer-aided milling, and 3D printing (additive manufacturing) [12]. Each reflect not only geometric accuracy of the manufacturing process, but also polymer-level material behaviour, with unique advantages and limitations: Milling involves the subtractive shaping of pre-polymerized PEEK blanks using CAD/CAM systems. It offers high precision and repeatability but may suffer from tool access limitations and material wastage [13–15]. Pressing melts and injects PEEK granules into molds under heat and pressure, a process adapted from metal-free ceramic techniques. It can produce dense restorations but is vulnerable to polymer shrinkage and cooling distortions [16, 17]. 3D PEEK Printing, particularly Filament deposition modeling (FDM) or selective laser sintering (SLS), enables mass customization and complex geometries while reducing waste. However, it often associated with layer-by-layer inconsistencies, porosities, and anisotropic mechanical behavior, variable degrees of crystallinity depending on thermal history which may compromise adaptation [18–21].

the Apium P400’s in-process infrared heating aims to increase interlayer fusion and crystallinity, which may reduce shrinkage and improve internal conformity. These material factors (crystallinity, anisotropy, and thermal contraction) therefore plausibly contribute to the observed group differences and required further study .

Despite the increasing use of these techniques, comparative data on the marginal and internal adaptation of implant-supported PEEK crowns across all three methods remain limited. Existing studies have largely focused on tooth-supported restorations or single-unit crowns, leaving a gap in evidence for implant-based applications [22, 23].

To overcome the limitations of traditional adaptation evaluation techniques, this study employs µCT, a non-invasive imaging modality that provides high-resolution, three-dimensional visualizations of gap dimensions and internal voids. µCT can detect discrepancies smaller than 50 μm, making it ideal for identifying clinically relevant deviations in crown gap [5, 24]. It allows volumetric analysis without compromising sample integrity, offering a superior method for comparing fabrication techniques [25, 26].

This study addresses a specific gap in the literature by providing a comparative µCT based evaluation of marginal and internal adaptation of PEEK crowns fabricated using three distinct manufacturing techniques (CAD-CAM milling, heat pressing, and FFF 3D printing). While existing studies on PEEK predominantly focus on mechanical properties, bonding performance, or veneering behavior, high-resolution, non-destructive µCT comparisons of dimensional accuracy across fabrication modalities remain limited. The null hypothesis was that no differences would exist in marginal or internal gaps among milled, pressed, and 3D-printed PEEK crowns. The alternative hypothesis anticipated that milling would yield the smallest discrepancies due to the inherent precision of subtractive manufacturing.

## Materials and methods

This in-vitro study evaluated the marginal and internal adaptation of implant-supported PEEK crowns fabricated using three different techniques: CAD-CAM milling, heat pressing, and 3D printing. The materials used in the study are listed in Table 1.

**Table 1:** Materials used in the study

Trade Name	Manufacturing	Composition	Fabrication Method	Lot No.
Zimmer Tapered Screw-Vent^®^ Implant System	Zimmer Biomet, USA	Titanium alloy (e.g., Ti-6Al-4 V)	CNC Milling / machined	ZTV-IMPL-0423-BX9
Contour Zirconia Abutments	Zimmer Biomet, USA	Zirconia	CAD/CAM Milling / machined	ZCA-0501-22KZ
breCAM. BioHPP	bredent GmbH	BioHPP (ceramic-reinforced PEEK)	CAD/CAM Milling	BHP-MIL-2307-A34
BioHPP Pellets	bredent GmbH	Ceramic-reinforced PEEK	Pressing (Hot-press injection)	BHP-PEL-2311-RX7
KATANA Wax	Kuraray Noritake Dental	Wax-based resin (CAD/CAM wax)	Milling (pattern creation)	KWX-MIL-2402-C15
FinoVest Phosphate-Bonded Investment	FINO GmbH, Germany	Phosphate-bonded refractory material	Investment Casting	FVP-CAST-0410-HG8
Medical-grade PEEK Filament	NA (e.g., Evonik, Invibio)	Polyetheretherketone (PEEK)	3D Printing (FDM/FFF)	PEEK-FDM-2404-MN3

Sample size was calculated using G*Power software (Version 3.1.9.7), based on the effect size reported by Attia et al. [24]. A total of 24 specimens was needed to detect an effect size of F = 1.69 with 95% power and a significance level (α) of 0.05. To increase the reliability of the results and allow for possible specimen loss, the final sample size was increased to 30 crowns (*n* = 10 per group).

### Specimen preparation

Thirty implant-abutment assemblies were mounted in custom cylindrical acrylic resin blocks (30 mm × 20 mm) using clear auto-polymerizing acrylic resin (Orthoplast; Vertex, Zeist, The Netherlands).The implant-abutment assembly was measuring 30 mm × 20 mm. Each assembly consisted of a Zimmer Biomet implant (Certain^®^ internal hex, 4.5 mm diameter, 10 mm length; Zimmer Biomet, Warsaw, IN, USA) restored with a prefabricated zirconia abutment (Zimmer Biomet, Warsaw, IN, USA). The implant was positioned vertically using a parallelometer to ensure precise alignment, with the implant–abutment interface positioned 3 mm above the acrylic surface. Acrylic resin was added incrementally under vibration to minimize internal voids and reduce polymerization shrinkage.

The PEEK crowns were fabricated using three different techniques: Group MP (Milled PEEK): 10 crowns were milled from BioHPP PEEK disks (20 mm height, 98.5 mm diameter; bredent GmbH) using a 5-axis CAD-CAM milling machine (imes-icore 150 Pro).

Group PP (Pressed PEEK): 10 wax patterns **(**KATANA Wax Kuraray Noritake Dental, Japan) were first milled using the same CAD design as the MP group to ensure complete standardization of crown geometry and design parameters. These were invested in a silicone ring using phosphate-bonded investment material (Brevest; bredent medical GmbH). After burnout (630 °C for 90 min), medical-grade PEEK pellets were heat-pressed into the mold using a vacuum pressing machine (For 2 press; bredent medical GmbH) at 400 °C and 0.4 MPa pressure.

Group 3DP (3D Printed PEEK): Ten crowns were fabricated using fused filament fabrication (FFF) with a medical-grade PEEK filament (VESTAKEEP^®^i4 3DF, Evonik) and an FFF printer (Apium P400; Apium Additive Technologies GmbH). The crown design was performed in exocad DentalCAD (exocad GmbH) and exported as an STL file. Slicing and g-code generation for the Apium P400 printer were performed using the Apium P400’s integrated/manufacturer-recommended slicing software (Apium’s printer control software), with the following printing parameters: nozzle temperature ~ 480 °C, build-plate temperature 240 °C, layer height 0.20 mm, print speed 35 mm/s, and a raft support strategy. The Apium P400 was used as the fabrication platform (Apium Additive Technologies GmbH).

The digital model was converted into an STL file and sliced using Dental CAD (exocad GmbH) for FFF 3D printing. Medical-grade pure PEEK filament was extruded at ~ 480 °C through a 0.4 mm nozzle, moving at 35 mm/s over a 240 °C heated carbon fiber bed. Layers (0.2 mm thick) were deposited sequentially with strong adhesion. The Apium P400 printer used infrared in-process annealing to enhance mechanical strength without prolonging print time. A raft support strategy prevented warping or delamination, and strict contamination control was maintained by sanitizing and dedicating equipment solely to PEEK processing, ensuring efficient and precise fabrication of high-performance components.

Printing parameters for the Apium P400 (Apium Additive Technologies GmbH) were: nozzle temperature 480 °C, build-plate temperature 240 °C, layer height 0.20 mm, print speed 35 mm/s, nozzle diameter 0.4 mm, infill: 100% (solid), raster (infill) orientation: alternating 0°/90° per layer, perimeter (shell) count: 2, and no active part-cooling fan (cooling disabled) to promote interlayer bonding. The printer’s in-process infrared annealing (adaptive heating) was enabled as provided by the Apium P400 platform to enhance interlayer crystallinity; no additional post-print thermal annealing step was applied. A raft support strategy was used to minimize warping.

**Fig. 1:**
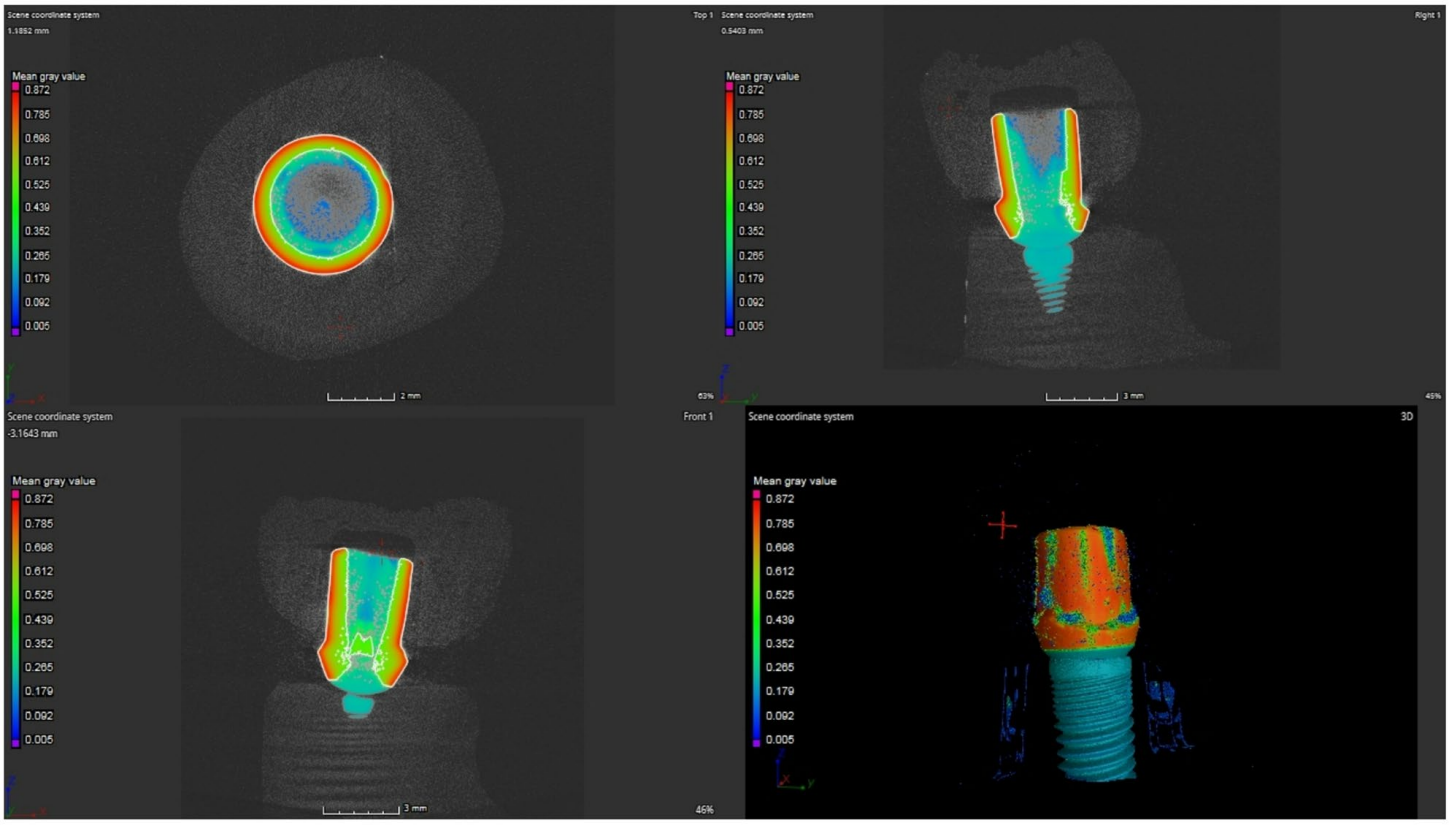
µCT analysis for different aspects

After printing/milling/pressing, crowns underwent only support/raft removal and gentle cleaning. Each crown was seated onto its corresponding zirconia abutment with gap-checking material (Fit Checker II; GC Corp), steam-cleaned for 10 s, and air-dried for 15 min, no additional contouring, grinding, or polishing of the margin was performed. Marginal and internal adaptation was assessed using µCT, a non-destructive and highly accurate 3D imaging modality (Fig. 1) [3, 5–7]. µCT scans were performed using a high-resolution scanner (Nikon XTH 225 ST; Nikon Metrology Ltd.), calibrated with a ruby artifact. Scanning parameters included: 170 kV, 7.6 W, 4000 ms exposure, 250-µm copper filter, and voxel size of 9.2 μm. The µCT-CT system was calibrated before each scanning session using a certified ruby artifact, in accordance with the manufacturer’s recommendations. This ensured geometric accuracy and consistency of the voxel size (9.2 μm) across all scans. The reconstructed 3D volumes were analyzed using VGStudio MAX 3.4 software (Volume Graphics GmbH). To ensure measurement reproducibility, a subset of specimens was re-analyzed by two independent examiners, demonstrating high reliability (ICC > 0.92). A uniform ISO 50% threshold was applied for all scans. A single calibrated examiner (M.M.) conducted all measurements. Cross-sectional slices were obtained in sagittal and coronal planes, with 12 standardized measurement points per crown.

To ensure reproducibility and standardization, each crown was seated onto its corresponding zirconia abutment using a customized setting machine applying a constant vertical load of 5 kg, the applied load was verified using calibrated dead weights before testing to ensure consistent seating conditions across all specimens. Importantly, no cement was used to secure the crown to the abutment during µCT examination to avoid any artifacts or illusions introduced by cement layers that could interfere with accurate gap measurements. The no luting agent concept was used to isolate and measure the dimensional accuracy inherent to each fabrication technique, without the confounding variables introduced by cement film thickness or hydraulic seating effects.

**Fig. 2:**
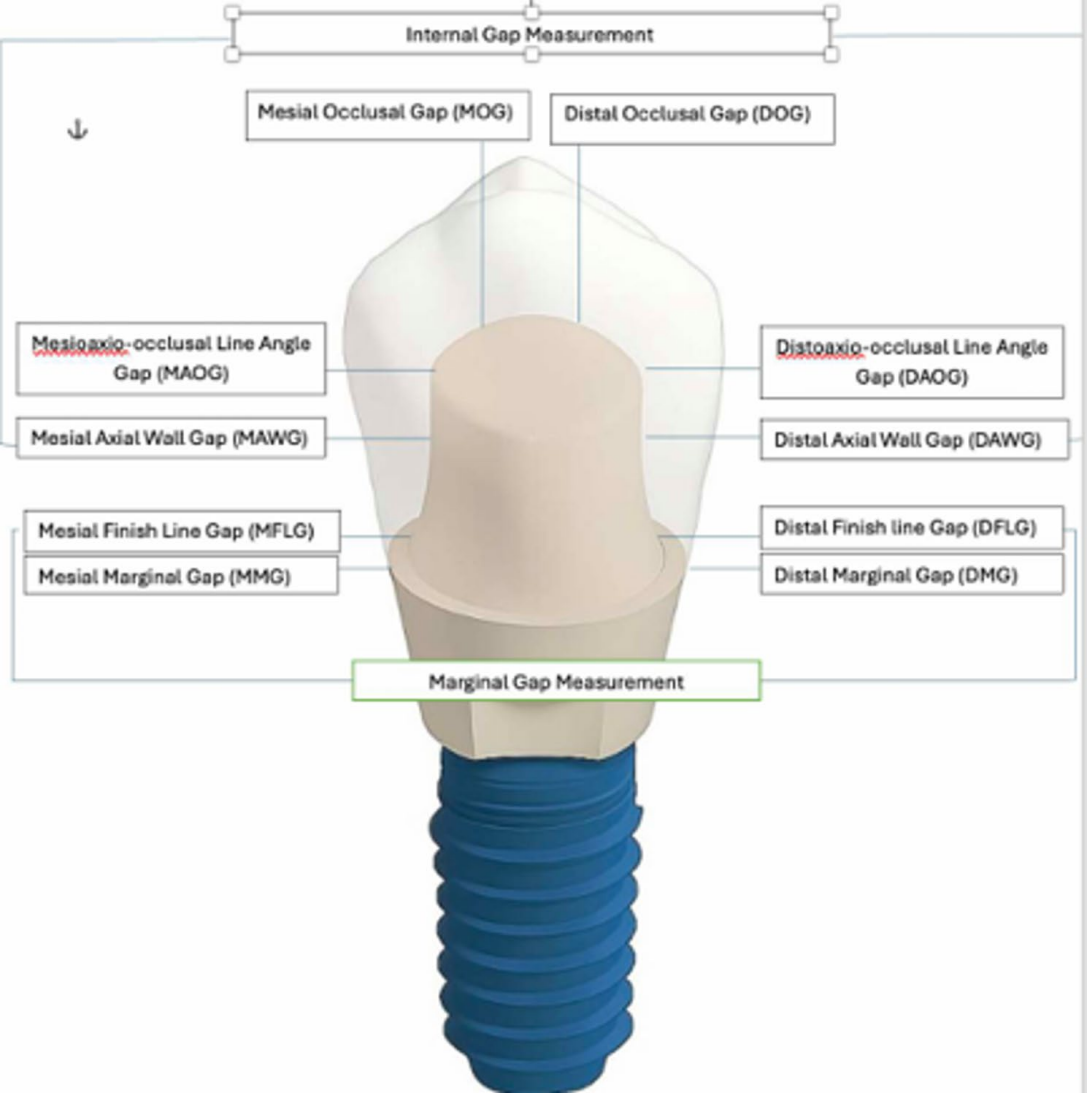
Schematic illustrations of measurement locations along the sagittal axis

**Fig. 3:**
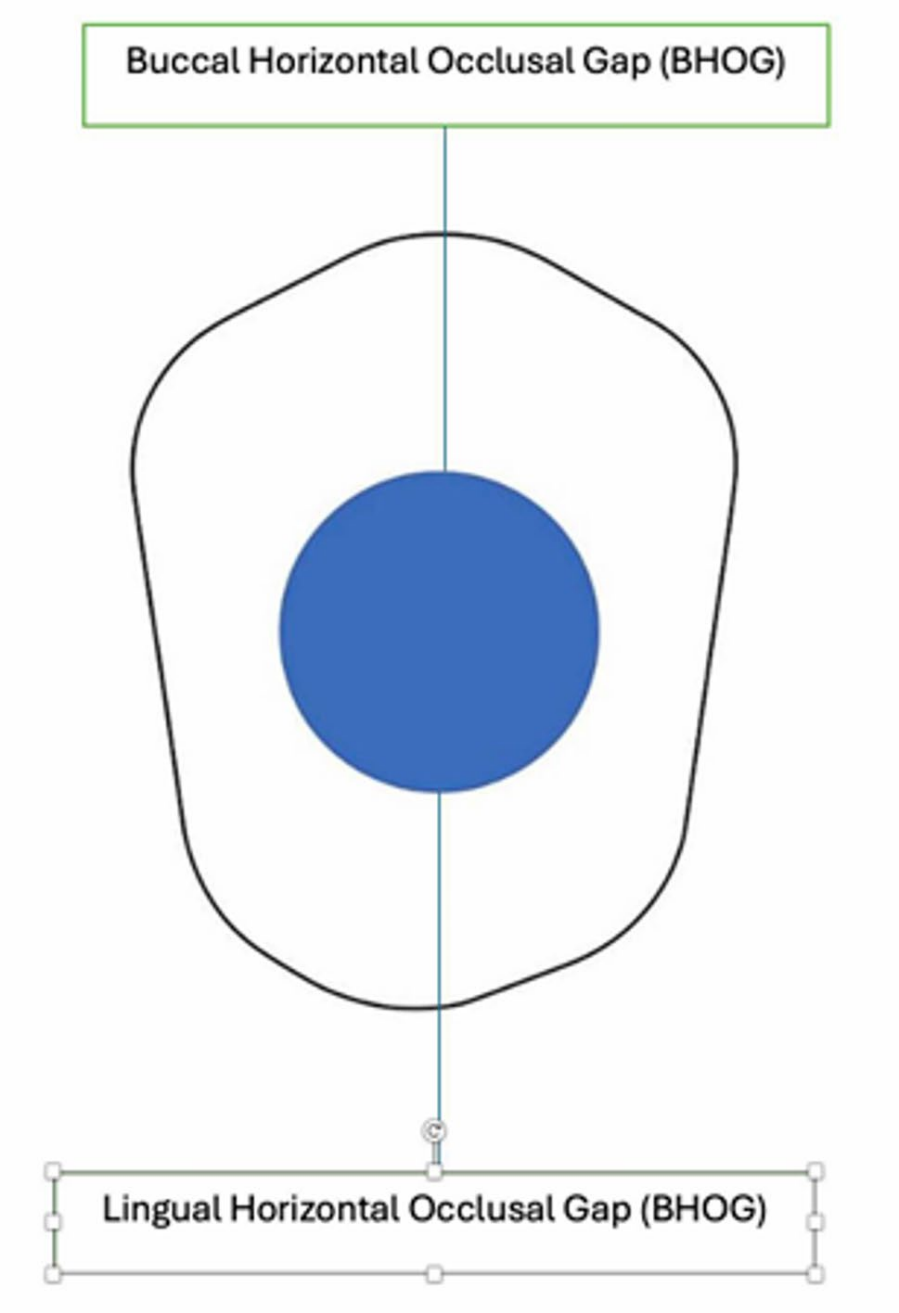
Schematic illustrations of measurement locations along the coronal axis

The evaluation of the crown adaptation included both marginal and internal gap measurements along the sagittal and coronal axes. In the sagittal axis, marginal gap parameters comprised the Mesial Marginal Gap (MMG), Mesial Finish Line Gap (MFLG), Distal Marginal Gap (DMG), and Distal Finish Line Gap (DFLG). Internal gap measurements in the same axis included the Mesial Axial Wall Gap (MAWG), Mesioaxio-Occlusal Line Angle Gap (MAOG), Mesial Occlusal Gap (MOG), Distal Axial Wall Gap (DAWG), Distoaxio-Occlusal Line Angle Gap (DAOG), and Distal Occlusal Gap (DOG). Additionally, internal adaptation along the coronal axis was assessed through the Buccal Horizontal Occlusal Gap (BHOG) and Lingual Horizontal Internal Gap (LHOG) (Figs. 2 and 3).

Numerical data were presented as mean and standard deviation (SD) values. They were tested for normality by examining the distribution and using the Shapiro-Wilk test. They were nonparametric and analyzed using the Kruskal-Wallis test and Dunn’s post hoc test. *P*-values were adjusted for multiple comparisons using the False Discovery Rate (FDR) method. The significance level was set at *P* < 0.05 within all tests. Effect size values were interpreted based on Cohen, J. (1992) (Cohen, J. (1992). A power primer. Psychological bulletin, 112(1), 155.) Statistical analysis was performed with R statistical analysis software version 4.4.2 for Windows [24].

## Results

**Fig. 4:**
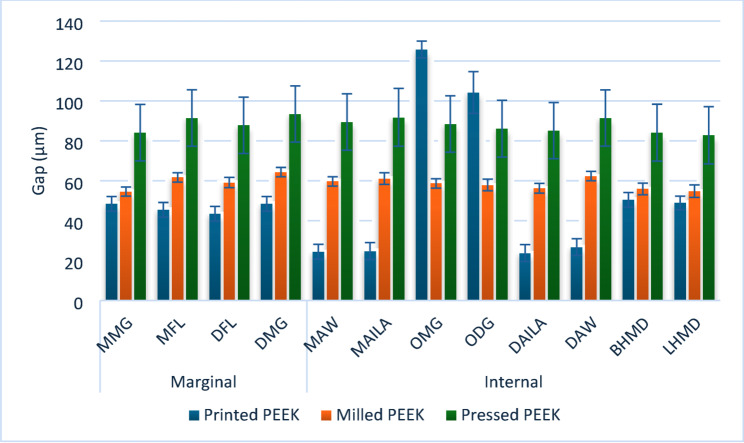
Bar chart showing mean and standard deviation values (error bars) for gaps measured at different points

**Fig. 5:**
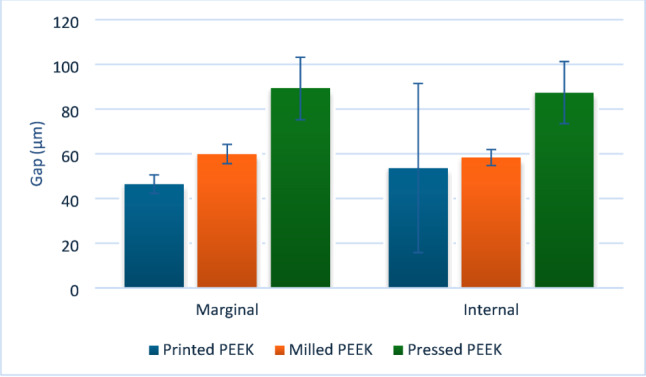
Bar chart showing mean and standard deviation values (error bars) for overall marginal and internal adaptation

Results of intergroup comparisons and summary statistics for different measured outcomes are presented in Table 2, Figs. 4 and 5.

**Table 2:** Intergroup comparisons

Measurement	Point	(Mean ± SD) (µm)			Test statistic	*P*-value	Rank based eta squared (95% CI)
		Printed PEEK	Milled PEEK	Pressed PEEK			
Internal gap	**MAW**	24.44 ± 3.68^C^	59.67 ± 2.35^B^	89.44 ± 14.16^A^	**23.15**	< 0.001*	0.88 (0.88 to 0.89)
	**MAILA**	24.78 ± 4.21^B^	61.11 ± 2.93^A^	91.78 ± 14.50^A^	**22.87**	< 0.001*	0.87 (0.82 to 0.89)
	**MOG**	125.78 ± 4.21^A^	58.67 ± 2.35^C^	88.44 ± 14.16^B^	**23.16**	< 0.001*	0.88 (0.88 to 0.89)
	**DOG**	104.22 ± 10.49^A^	57.89 ± 2.93^B^	86.11 ± 14.24^A^	**20.22**	< 0.001*	0.76 (0.66 to 0.89)
	**DAILA**	23.78 ± 4.21^C^	56.22 ± 2.44^B^	85.11 ± 14.10^A^	**23.16**	< 0.001*	0.88 (0.88 to 0.89)
	**DAW**	26.78 ± 4.21^C^	62.33 ± 2.35^B^	91.44 ± 14.09^A^	**23.16**	< 0.001*	0.88 (0.88 to 0.89)
	**BHMD**	50.44 ± 3.68^B^	55.89 ± 2.93^B^	84.11 ± 14.24^A^	**20.66**	< 0.001*	0.78 (0.68 to 0.88)
	**LHMD**	48.89 ± 3.41^B^	54.78 ± 3.15^B^	82.78 ± 14.32^A^	**21.10**	< 0.001*	0.80 (0.69 to 0.89)
	**MMG**	48.44 ± 3.68^B^	54.56 ± 2.30^B^	84.11 ± 14.12^A^	**21.46**	< 0.001*	0.81 (0.72 to 0.89)
	**Internal gap**	53.64 ± 37.83^B^	58.32 ± 3.57^B^	87.40 ± 13.88^A^	**85.41**	< 0.001*	0.88 (0.88 to 0.89)
Marginal gap	**MFL**	45.44 ± 3.68^C^	61.67 ± 2.35^B^	91.44 ± 14.16^A^	**23.15**	< 0.001*	0.88 (0.88 to 0.89)
	**DFL**	43.44 ± 3.68^C^	59.11 ± 2.57^B^	87.78 ± 14.13^A^	**23.15**	< 0.001*	0.88 (0.88 to 0.89)
	**DMG**	48.44 ± 3.68^C^	64.33 ± 2.35^B^	93.44 ± 14.09^A^	**23.15**	< 0.001*	0.88 (0.88 to 0.89)
	**Marginal gap**	46.44 ± 4.12^C^	59.92 ± 4.31^B^	89.19 ± 13.98^A^	**91.36**	< 0.001*	0.87 (0.82 to 0.89)

values with different superscripts within the same horizontal row are significantly different

*CI* Confidence Interval

* significant (*P* < 0.05)

All comparisons at different points were statistically significant and showed large effect sizes. For internal gap measurements at MAW, DAILA, and DAW, pairwise comparisons showed that gaps were largest in pressed samples, followed by milled samples, and smallest in printed samples (i.e., the best adaptation). At MAILA, pressed and milled samples had significantly larger gaps than printed samples. At MOG, all comparisons were statistically significant, with the highest gap observed in pressed samples, followed by printed samples, while the best adaptation (i.e., the smallest gap) was measured in milled samples. Measurements at DOG revealed that gaps in both printed and pressed samples were significantly larger than in milled samples. For the overall internal gap and measurements at BHMD and LHMD, pressed samples exhibited significantly larger gaps compared to printed and milled samples.

For marginal gaps, measurements taken at MMG revealed that pressed samples had significantly larger gaps than printed and milled samples. For other marginal measurements (MFL, DFL, DMG), including the overall marginal gap, all comparisons were statistically significant, with pressed samples showing the largest gaps, followed by milled samples, while the best adaptation (i.e., smallest gaps) was observed in printed samples.

## Discussion

High-resolution µCTimaging was employed to evaluate the influence of CAD-CAM milling, heat pressing, and 3D printing on the marginal and internal adaptation of implant-supported PEEK crowns. Differences in adaptation were observed among the three fabrication techniques, with each method demonstrating region-specific performance characteristics. While all groups achieved clinically acceptable marginal adaptation (< 120 μm), statistically significant variations were detected across all measured sites, underscoring the critical role of fabrication technique in achieving precise adaptation for PEEK restorations.

µCT was selected for this study due to its well-established accuracy, reproducibility, and non-destructive nature [7, 27]. Unlike conventional two-dimensional evaluation methods, such as the silicone replica technique or direct microscopic measurements, which are susceptible to material distortion, shrinkage, and sectioning artifacts, µCT enables comprehensive three-dimensional volumetric analysis of restoration adaptation without compromising specimen integrity [5, 7]. The fine voxel resolution of 9.2 μm allowed precise detection of marginal and internal gaps at multiple critical sites, providing reliable quantitative data for comparative assessment across fabrication techniques. Although µCT involves higher operational costs and longer scanning times, its ability to perform repeated non-invasive measurements ensured consistent and reproducible evaluation throughout the study [7, 27].

Zirconia abutments were used because of their favorable optical and biomechanical properties. Their radiopacity enabled clear µCT differentiation between the crown, abutment, and air spaces, while eliminating metal-related artifacts commonly associated with titanium abutments, thereby enhancing measurement accuracy [25, 28, 29]. In addition, zirconia’s tooth-like color and high mechanical strength support both esthetic and functional reliability in implant prosthodontics.

For standardization, a 30 μm die spacer was applied to all crowns, beginning 1 mm above the finish line. This design ensured adequate space for luting material without compromising marginal adaptation. Importantly, marginal and internal adaptation were assessed without cementation to eliminate variability related to cement film thickness, seating pressure, and hydraulic effects [6, 28]. This approach is consistent with previous µCTstudies aiming to isolate the inherent dimensional accuracy of fabrication techniques rather than the confounding influence of cementation, which has been shown to increase marginal discrepancies [30–32].

The null hypothesis, which proposed that the fabrication technique would not influence the marginal and internal adaptation of implant-supported PEEK crowns, was rejected. Statistically significant differences were identified among CAD-CAM milled, 3D-printed, and heat-pressed crowns, indicating that the manufacturing workflow has a measurable effect on restoration adaptation.

The enhanced marginal adaptation observed in CAD-CAM milled and 3D-printed PEEK crowns is likely attributable to the precision of digital workflows, accurate margin detection, and continuous advancements in CAD-CAM technology [23, 33]. Specifically, milled PEEK crowns demonstrated superior adaptation at the occlusal surface (Table 2), exhibiting significantly smaller occlusal gaps compared with the other fabrication techniques (*P* < 0.001). This finding is consistent with the inherent accuracy of subtractive manufacturing, which minimizes dimensional changes associated with polymerization or cooling shrinkage and allows controlled material removal from industrially fabricated blocks [23, 34, 35].

In contrast, 3D-printed PEEK crowns showed the most favorable adaptation along axial walls and internal line angles (Table 2, Fig. 4). This may be attributed to the layer-by-layer fabrication process, which enables intimate conformity to the abutment’s axial surfaces [36]. Although additive manufacturing may be challenged in reproducing fine occlusal details compared with milling, its ability to achieve superior internal conformity is noteworthy. Nevertheless, the final accuracy of 3D-printed restorations remains highly dependent on printing parameters, material properties, and post-processing procedures, warranting further investigation and optimization [9, 12].

Heat-pressed PEEK crowns consistently demonstrated the largest marginal and internal discrepancies across most regions (Table 2). These findings likely reflect the inherent limitations of the heat-pressing technique, including thermal expansion and contraction during pressing and cooling cycles, as well as susceptibility to dimensional inaccuracies compared with digitally controlled fabrication methods [5, 24]. The multi-step, analog nature of the pressing process—similar to the conventional lost-wax technique—introduces additional sources of error, particularly those related to investment expansion and technician-dependent variables [23, 24]. This interpretation is consistent with previous studies reporting superior retention and adaptation of milled telescopic crowns compared with restorations fabricated using conventional techniques (Figs. 4 and 5) [14, 30].

The present findings are further supported by Attia et al., who reported mean marginal gap values of 45 ± 6 μm for milled PEEK copings and 92 ± 3 μm for pressed PEEK copings, confirming significantly better marginal and internal adaptation for milled restorations [23, 24].

Despite ongoing debate regarding whether CAD-CAM fabrication yields superior marginal adaptation compared with conventional techniques, marginal discrepancies below 120 μm are generally considered clinically acceptable [4, 27]. In contrast, acceptable thresholds for internal adaptation remain poorly defined and inconsistently reported in the literature [29]. The present results emphasize that while marginal adaptation may fall within clinically tolerable limits across different fabrication techniques, variations in internal adaptation may have important clinical implications.

A well-adapted internal surface facilitates proper seating of the restoration, minimizes stress concentration at the abutment–implant interface, and enhances retention and biomechanical stability [32, 37]. The observed region-specific differences suggest that fabrication technique selection should be guided by clinical priorities—favoring CAD-CAM milling when occlusal precision is critical and 3D printing when optimal internal conformity is desired.

This study has several limitations. Only one brand of PEEK material and a standardized zirconia abutment were evaluated; therefore, the results may vary with different material formulations, abutment designs, or implant connections. The use of standardized rather than patient-specific abutments may also limit direct clinical extrapolation. Future investigations should explore the influence of different PEEK grades, abutment configurations, implant systems, and cementation protocols on restoration adaptation. Additionally, in vivo studies are required to assess the long-term clinical behavior and longevity of PEEK crowns fabricated using different manufacturing techniques.

Within the limitations of this study, it can be concluded that the fabrication technique has a significant influence on the marginal and internal adaptation of implant-supported PEEK crowns. CAD-CAM milled crowns demonstrated superior occlusal adaptation, whereas 3D-printed crowns exhibited enhanced internal conformity along axial surfaces. Heat-pressed PEEK crowns showed the poorest overall adaptation. These findings highlight the importance of selecting fabrication workflows based on specific clinical objectives and support the continued optimization of digital manufacturing techniques to improve the predictability and longevity of PEEK-based implant restorations.

## Conclusion

Fabrication technique significantly affects the adaptation of implant-supported PEEK crowns. While all methods produced clinically acceptable margins, milled crowns showed the best occlusal adaptation, 3D-printed crowns achieved superior internal adaptation, and heat-pressed crowns performed the poorest. µCTproved valuable for precise, non-destructive evaluation. Clinically, choosing the right fabrication method is critical for enhancing prosthesis accuracy, implant longevity, and treatment success.

## Clinical significance

The accuracy of marginal and internal adaptation directly influences the biological and mechanical success of implant-supported crowns. This in vitro study evaluates adaptation by comparing measured gap values against the widely accepted clinical threshold of ~ 120 μm for marginal discrepancy. The findings, which show milling and 3D printing yield superior adaptation compared to heat pressing, can therefore guide clinicians toward fabrication methods that are more likely to enhance restoration longevity and reduce complications.

## Supplementary Information

Supplementary Material 1.

## Data Availability

The datasets used and/or analysed during the current study are available from the corresponding author on reasonable request.
