# Supplementary figures and images for: Evaluation of marginal and internal adaptation of implant-supported PEEK crowns fabricated by 3D printing, milling, and pressing: a micro-CT analysis
Source: BMC Oral Health. 2026 Apr 9;26:730. doi: 10.1186/s12903-026-08150-8 (PMC13109875; doi:10.1186/s12903-026-08150-8)

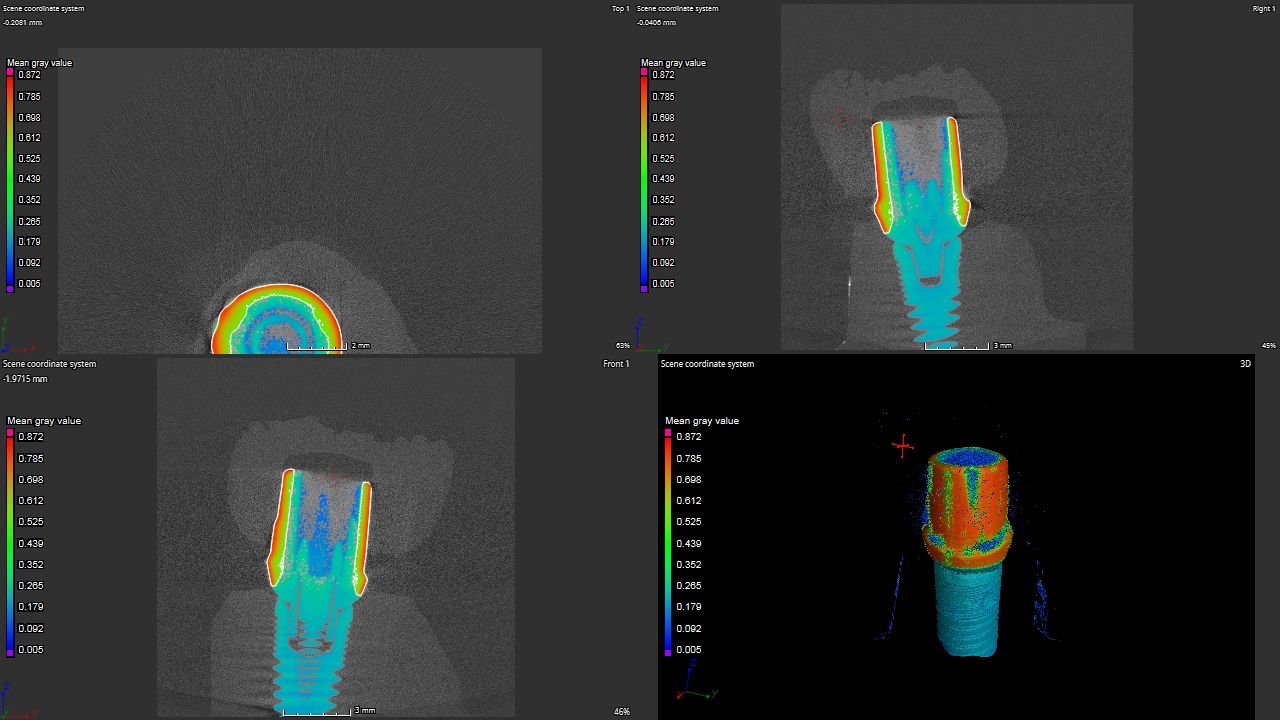

Supplement: Supplementary file 1 — Supplementary Material 1. [file 12903_2026_8150_MOESM1_ESM.zip › 13.jpeg]

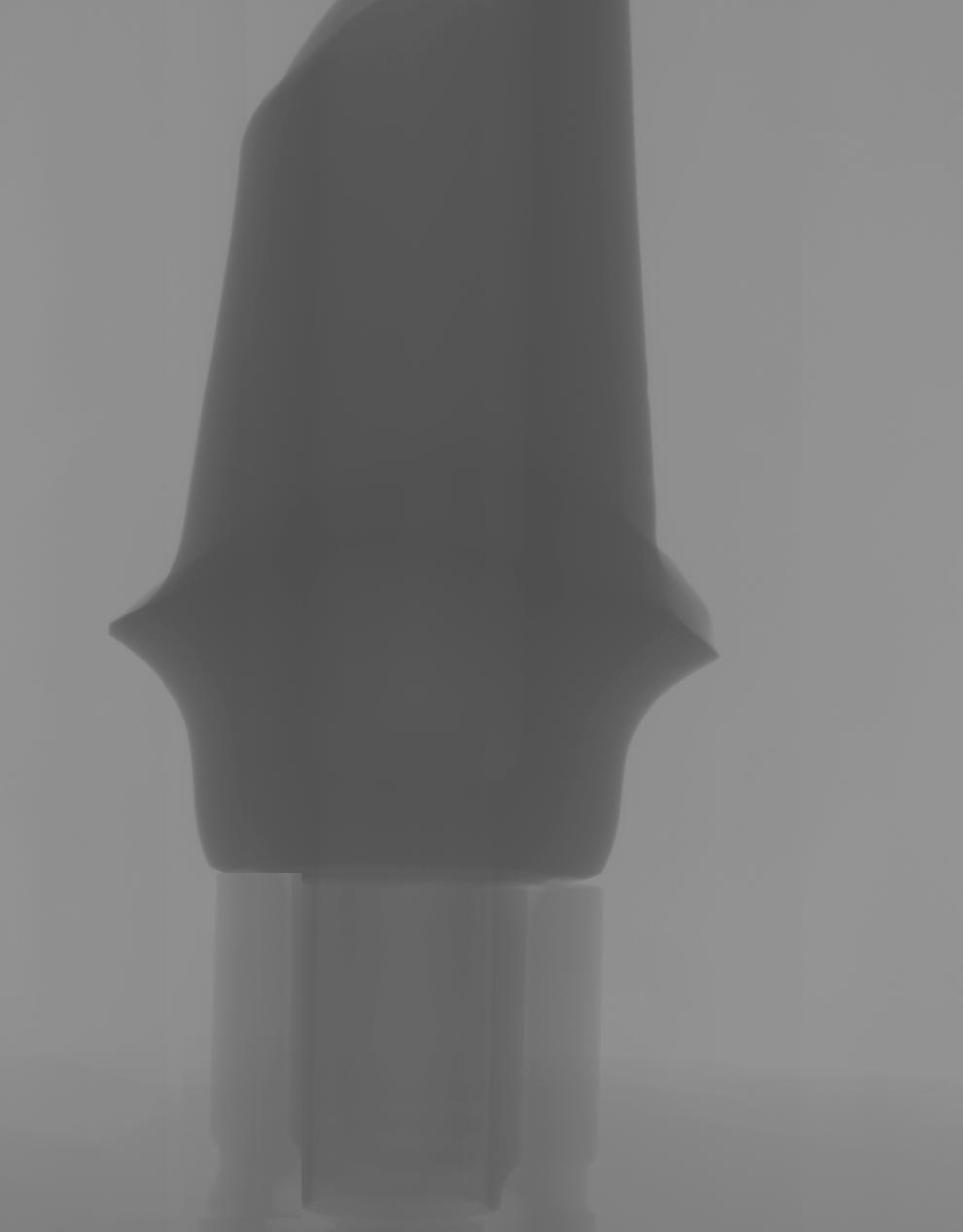

Supplement: Supplementary file 1 — Supplementary Material 1. [file 12903_2026_8150_MOESM1_ESM.zip › 3.jpeg]

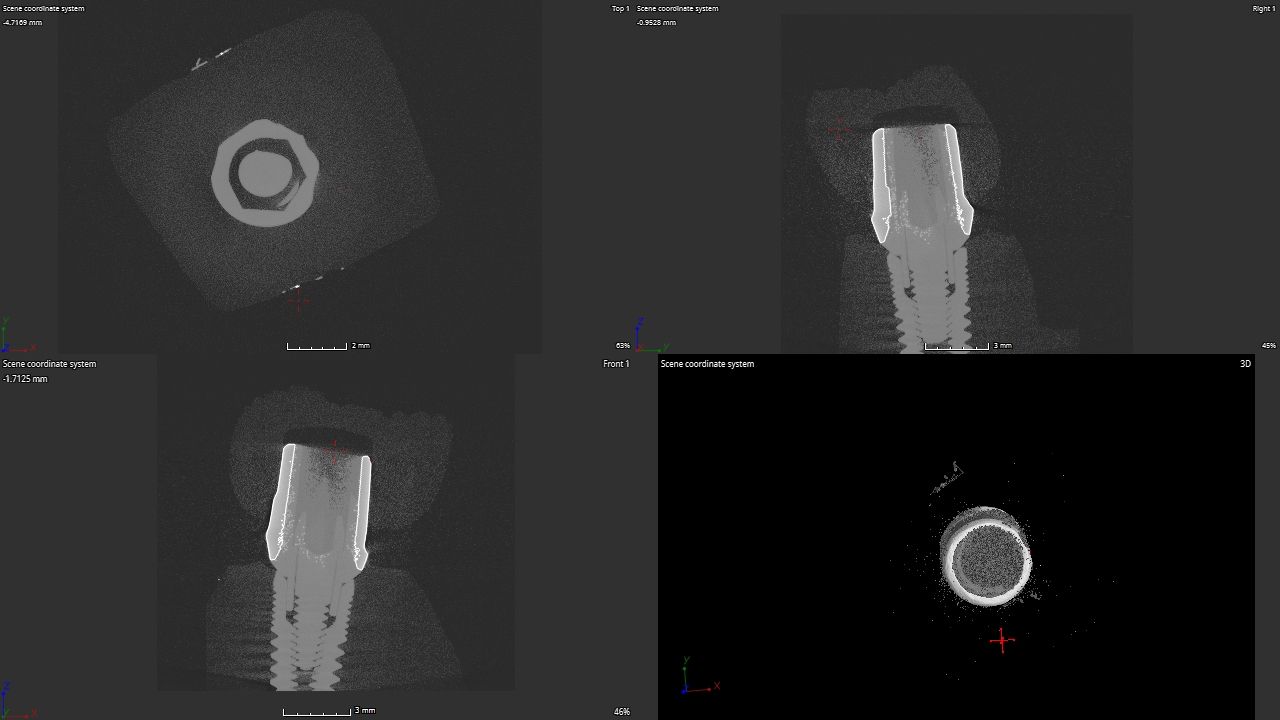

Supplement: Supplementary file 1 — Supplementary Material 1. [file 12903_2026_8150_MOESM1_ESM.zip › 10.jpeg]

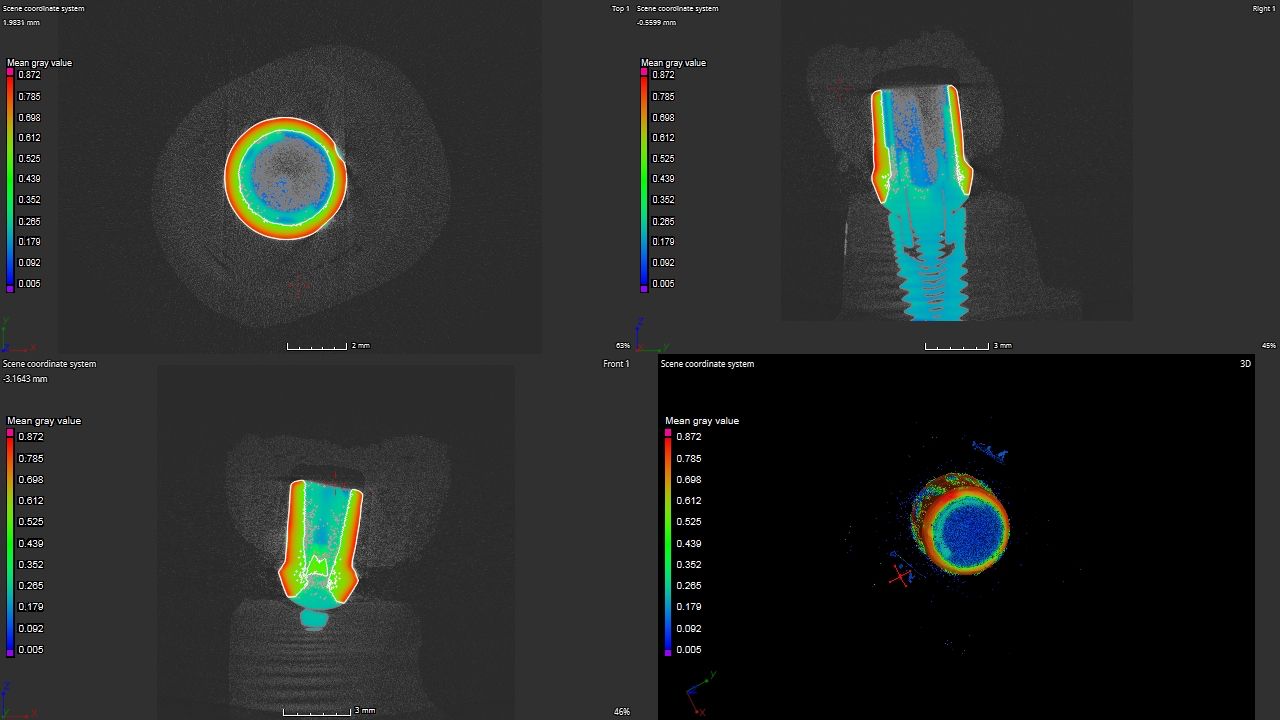

Supplement: Supplementary file 1 — Supplementary Material 1. [file 12903_2026_8150_MOESM1_ESM.zip › 17.jpeg]

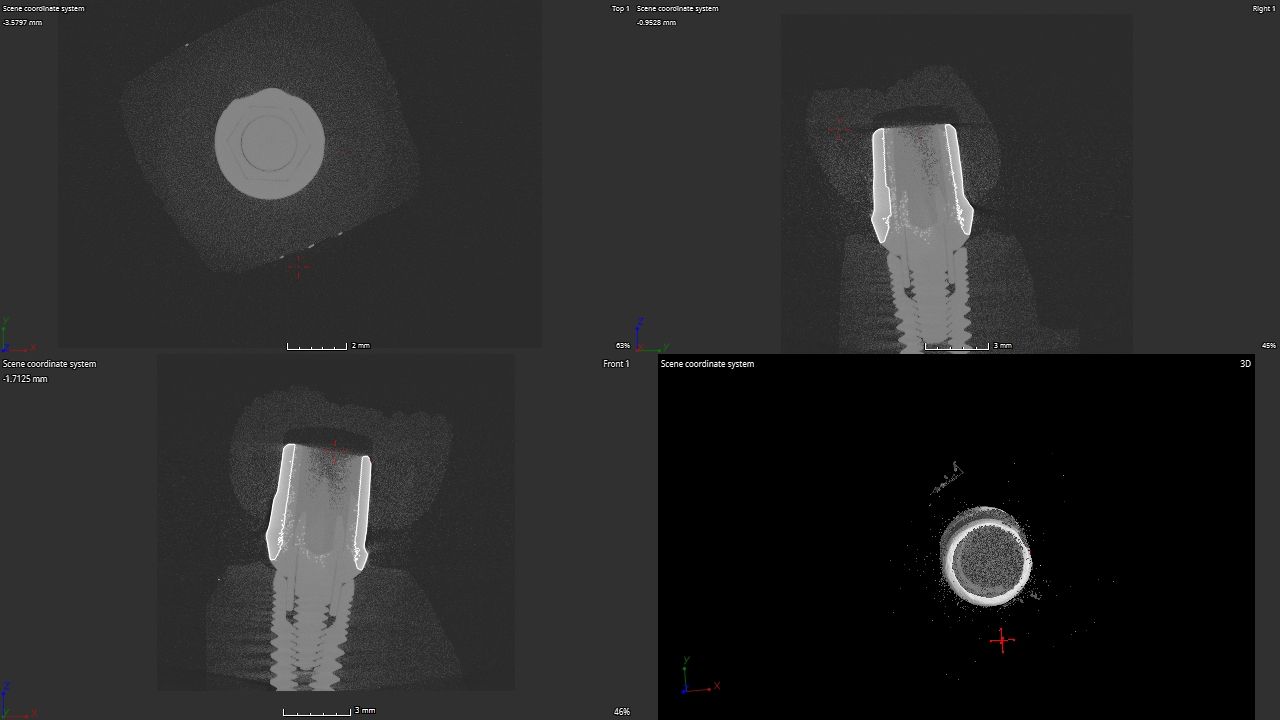

Supplement: Supplementary file 1 — Supplementary Material 1. [file 12903_2026_8150_MOESM1_ESM.zip › 19.jpeg]

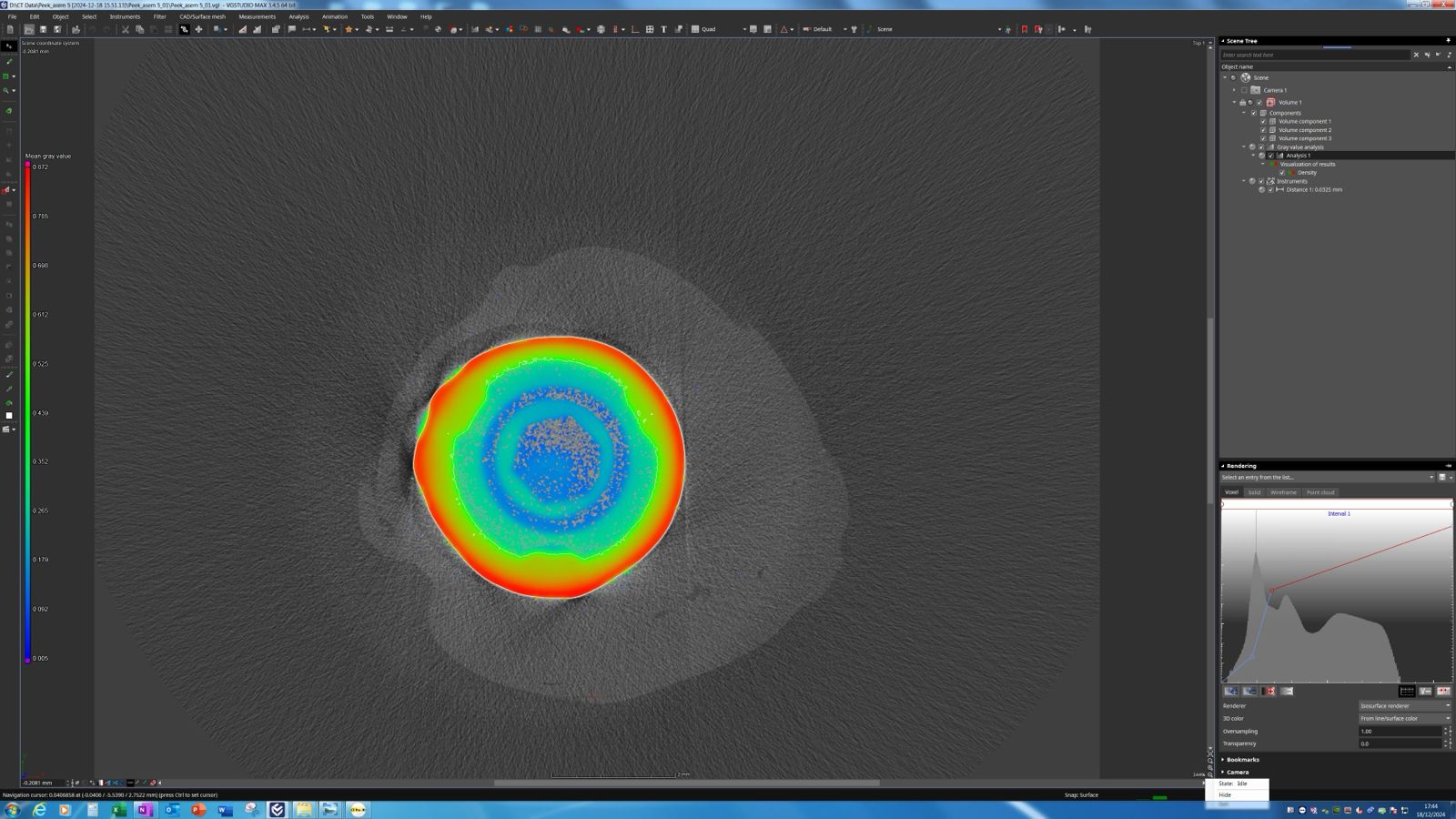

Supplement: Supplementary file 1 — Supplementary Material 1. [file 12903_2026_8150_MOESM1_ESM.zip › 14.jpeg]

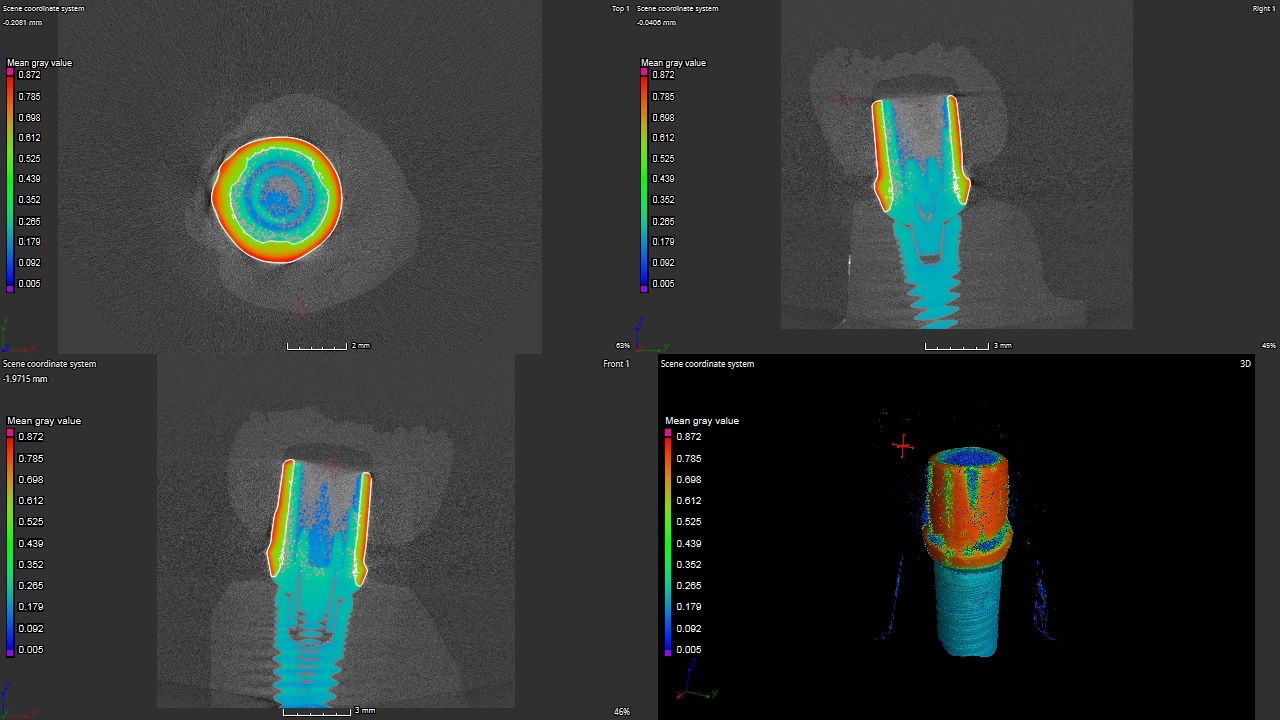

Supplement: Supplementary file 1 — Supplementary Material 1. [file 12903_2026_8150_MOESM1_ESM.zip › 15.jpeg]

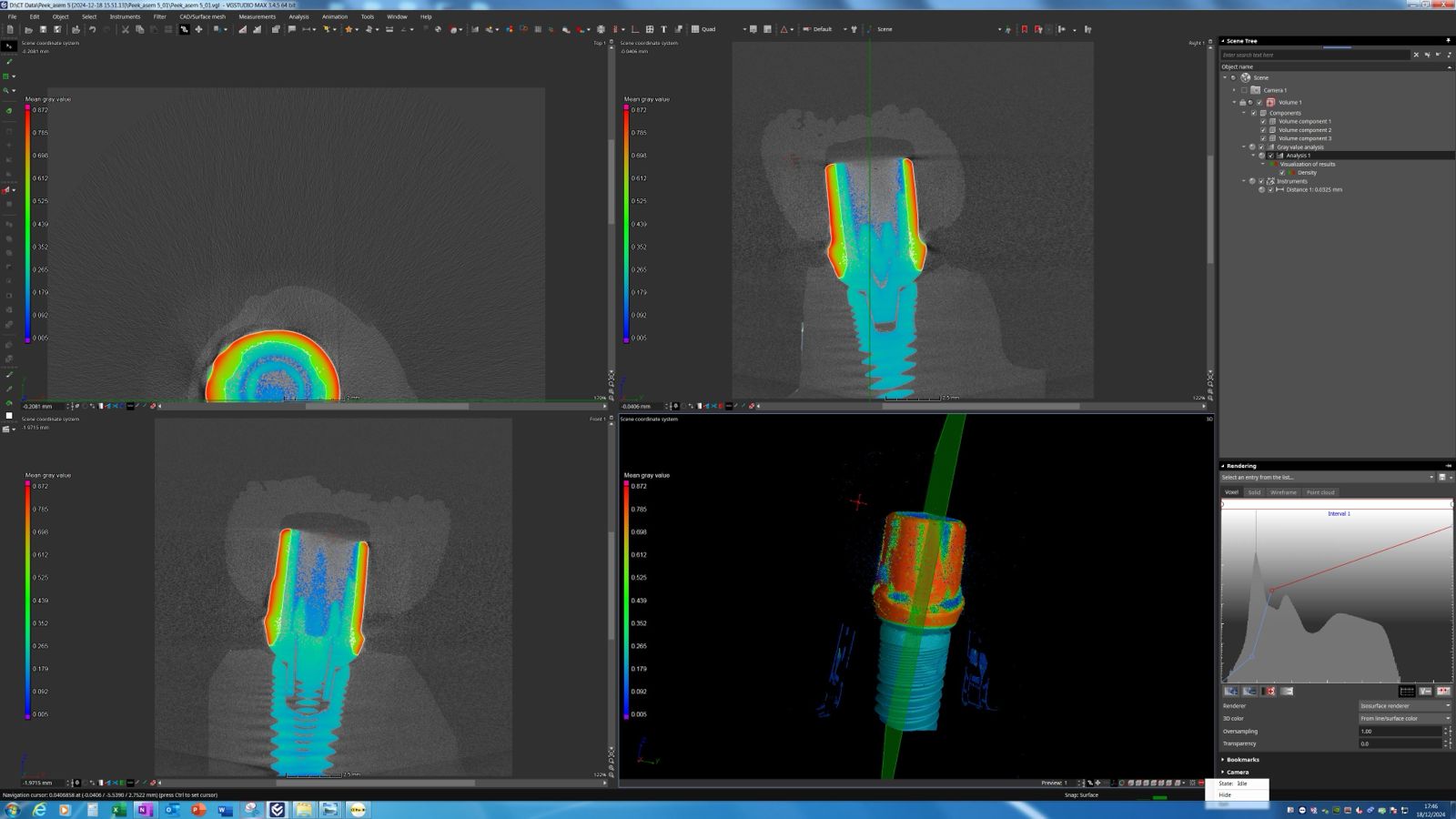

Supplement: Supplementary file 1 — Supplementary Material 1. [file 12903_2026_8150_MOESM1_ESM.zip › 16.jpeg]

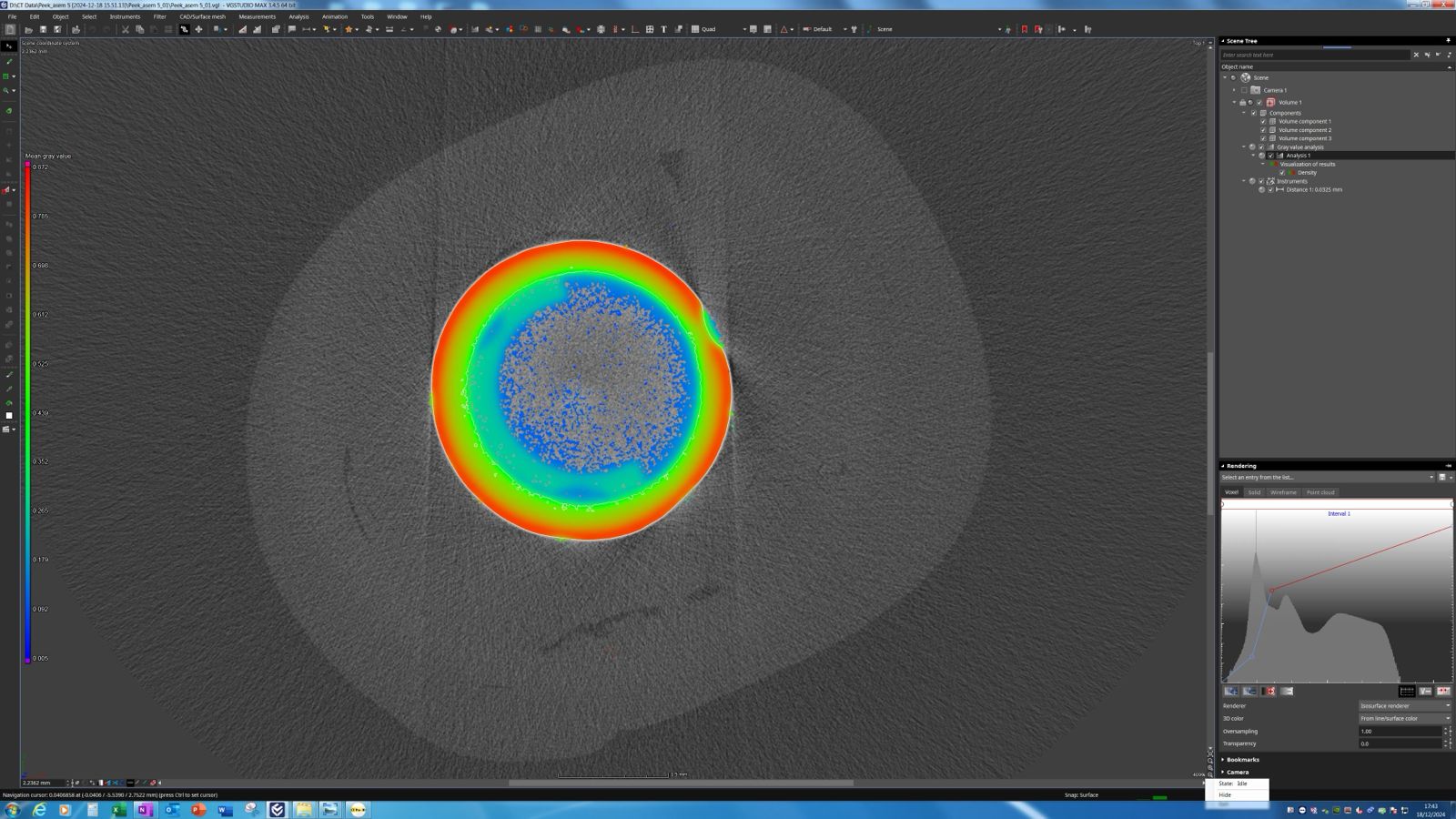

Supplement: Supplementary file 1 — Supplementary Material 1. [file 12903_2026_8150_MOESM1_ESM.zip › 18.jpeg]

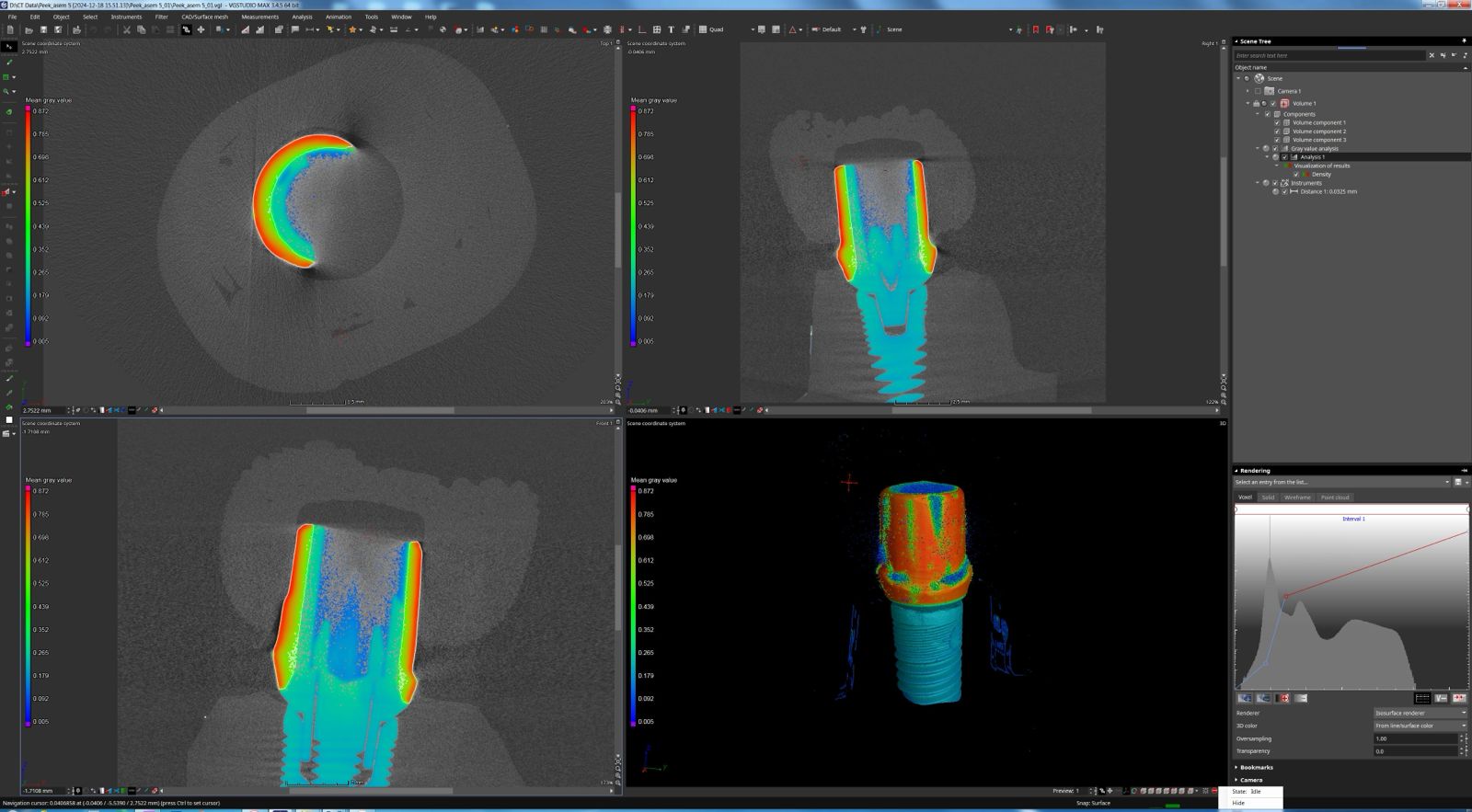

Supplement: Supplementary file 1 — Supplementary Material 1. [file 12903_2026_8150_MOESM1_ESM.zip › 20.jpeg]

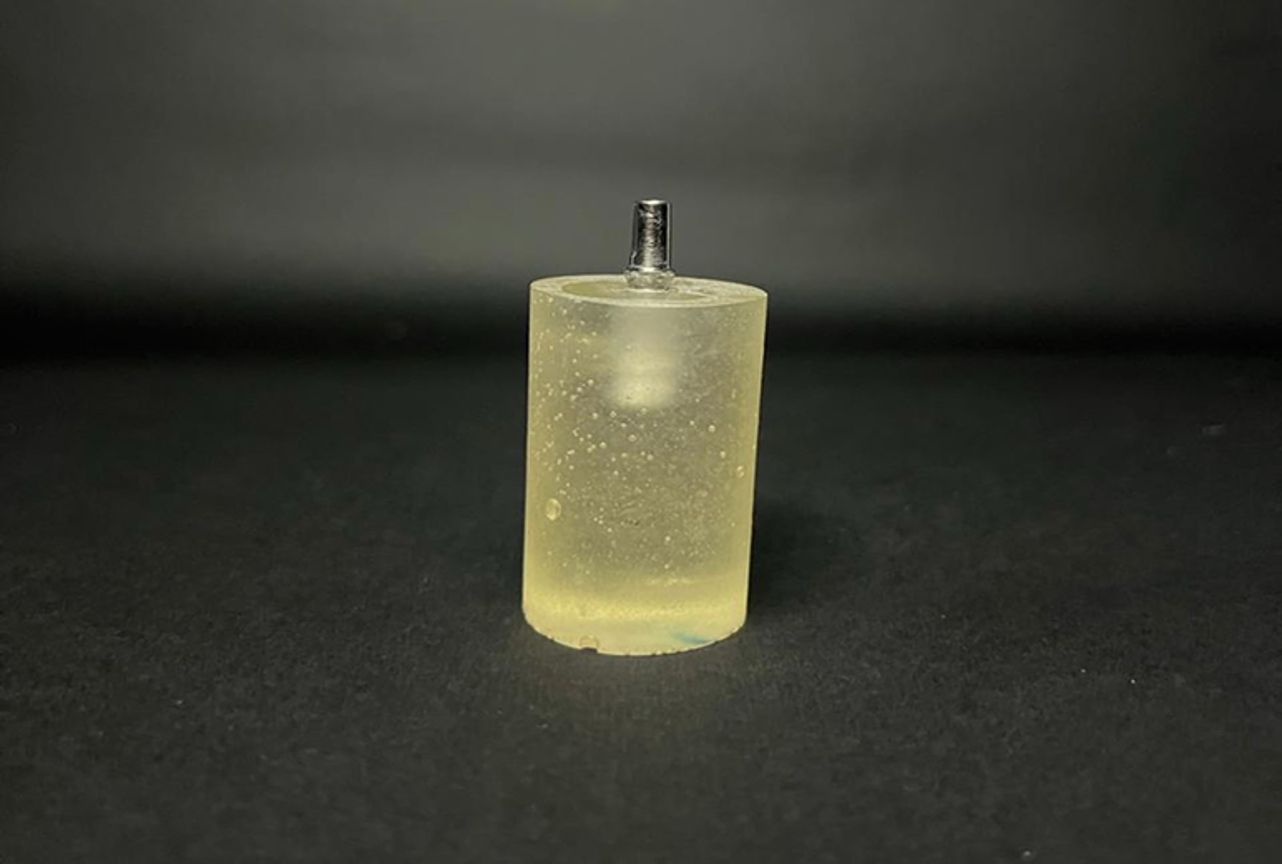

Supplement: Supplementary file 1 — Supplementary Material 1. [file 12903_2026_8150_MOESM1_ESM.zip › embedding 1.jpg]

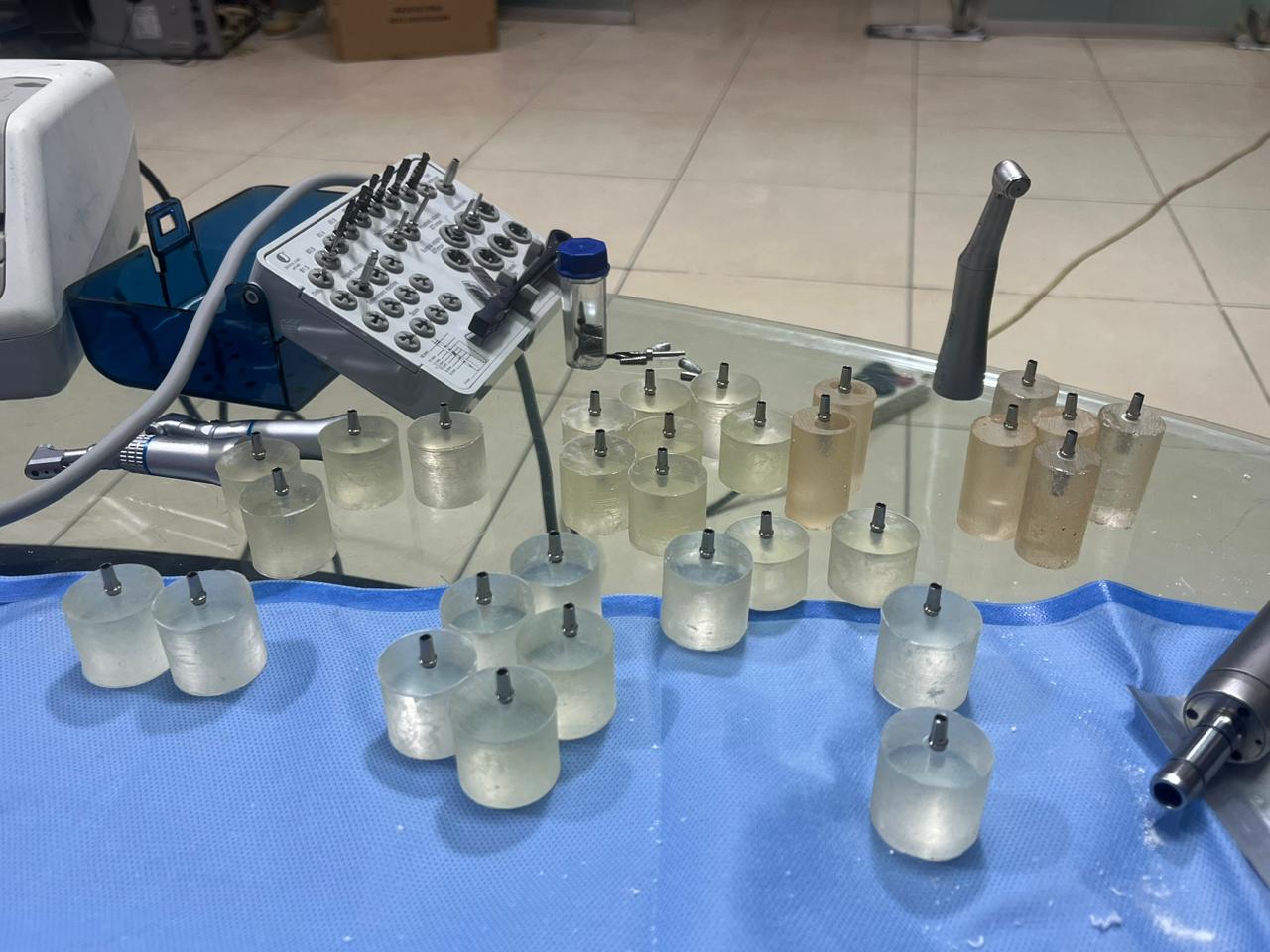

Supplement: Supplementary file 1 — Supplementary Material 1. [file 12903_2026_8150_MOESM1_ESM.zip › embedding 2.jpg]

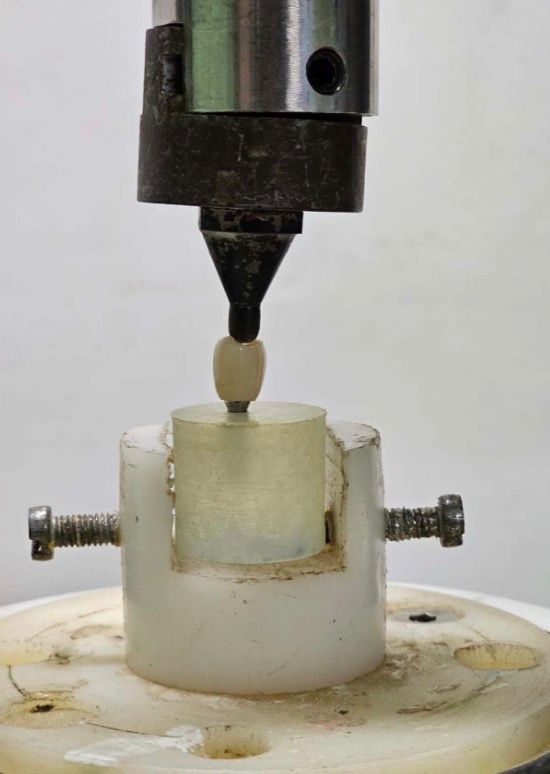

Supplement: Supplementary file 1 — Supplementary Material 1. [file 12903_2026_8150_MOESM1_ESM.zip › embedding.jpg]

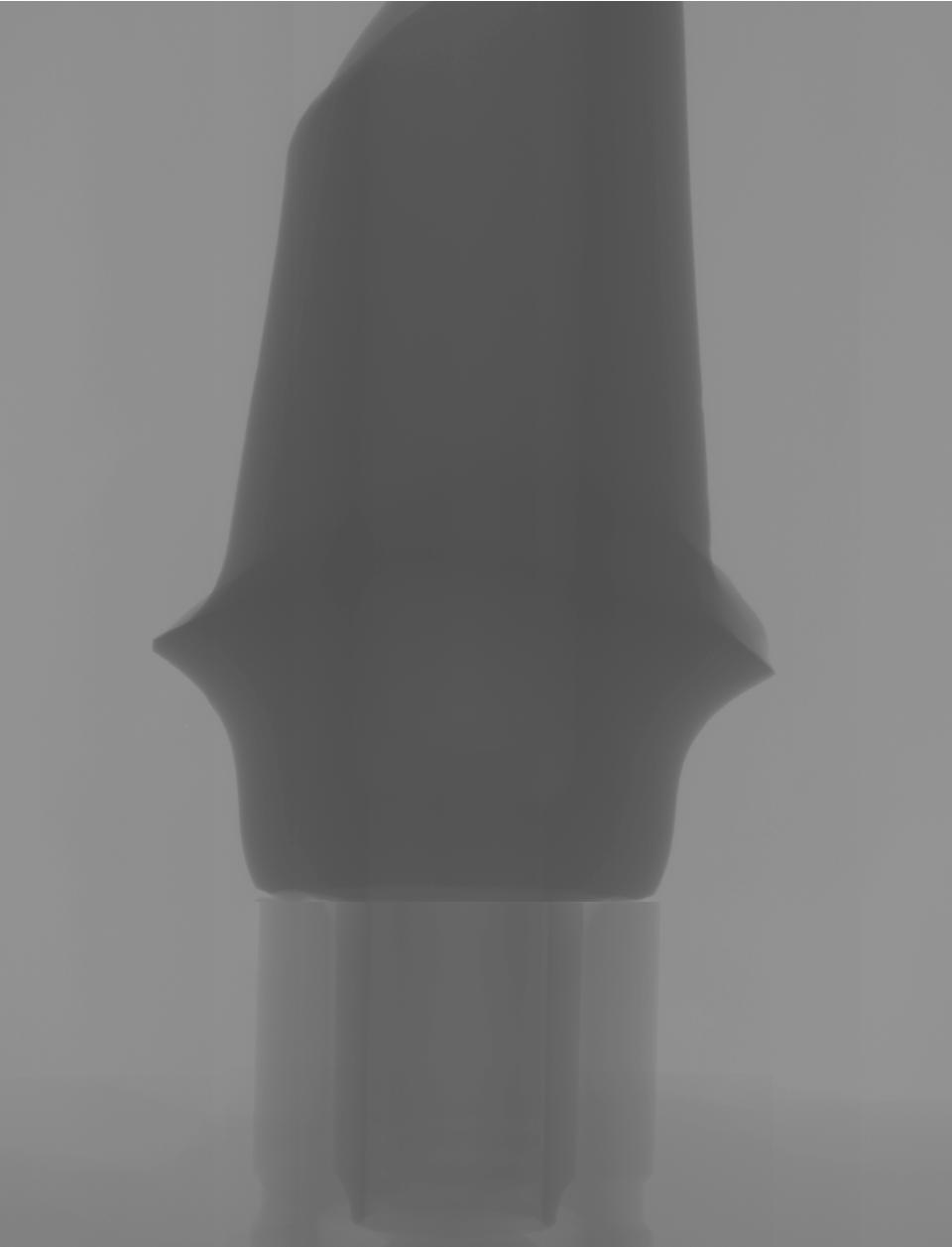

Supplement: Supplementary file 1 — Supplementary Material 1. [file 12903_2026_8150_MOESM1_ESM.zip › 1.jpeg]

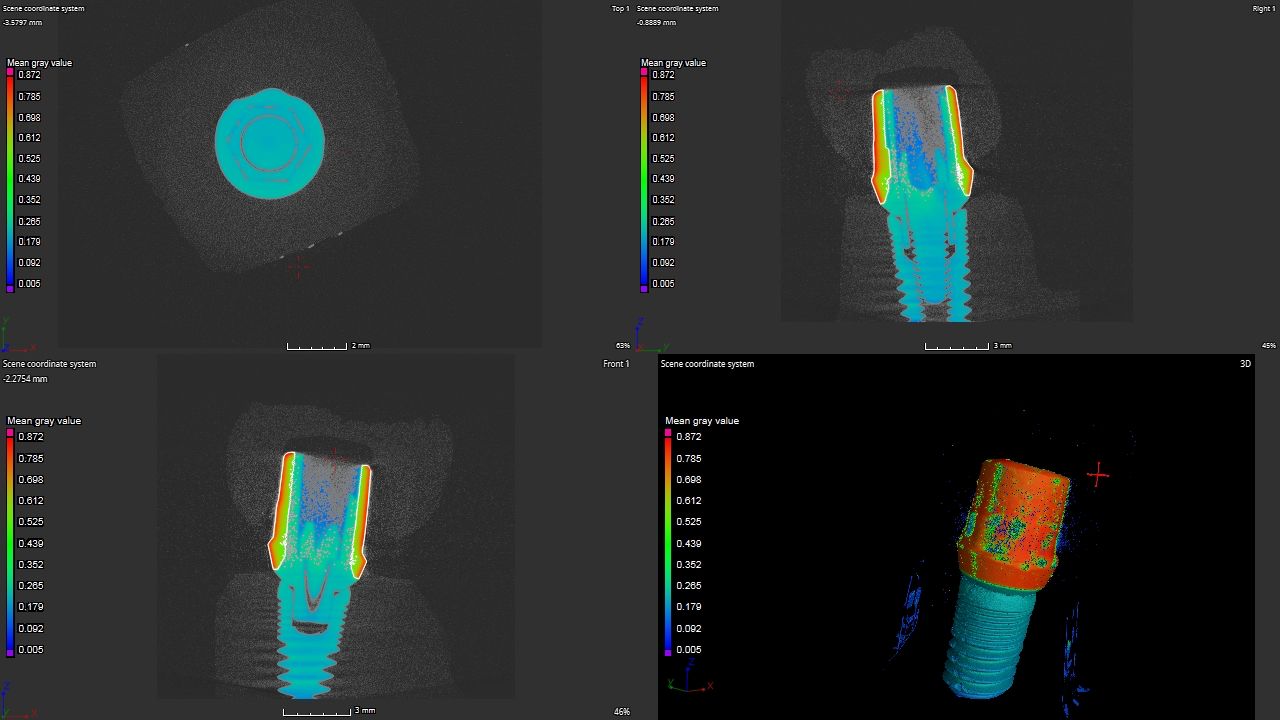

Supplement: Supplementary file 1 — Supplementary Material 1. [file 12903_2026_8150_MOESM1_ESM.zip › 2.jpeg]

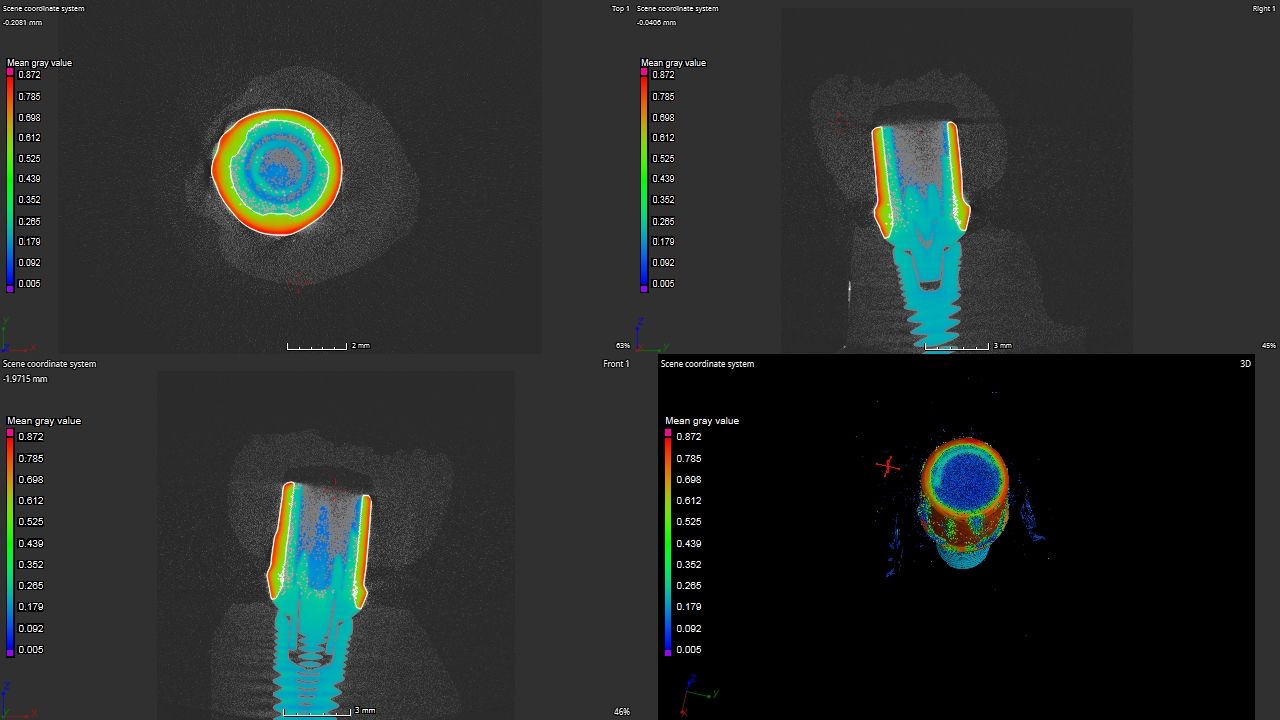

Supplement: Supplementary file 1 — Supplementary Material 1. [file 12903_2026_8150_MOESM1_ESM.zip › 4.jpeg]

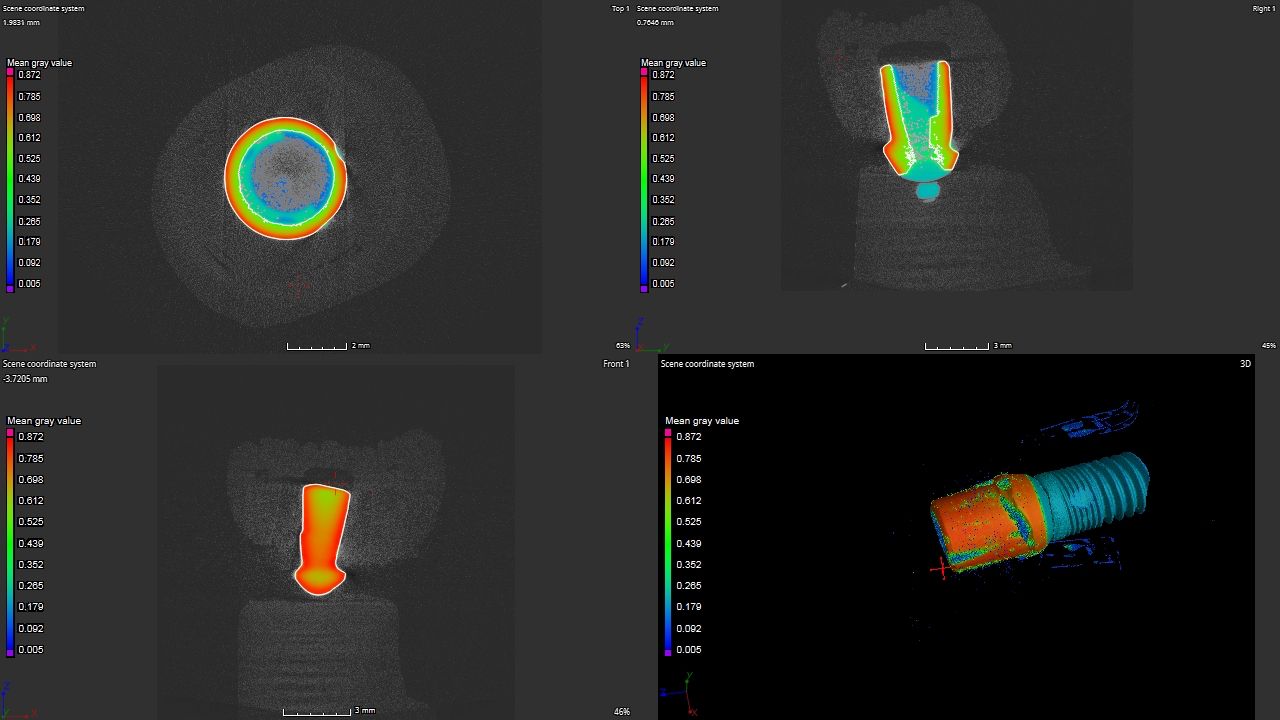

Supplement: Supplementary file 1 — Supplementary Material 1. [file 12903_2026_8150_MOESM1_ESM.zip › 5.jpeg]

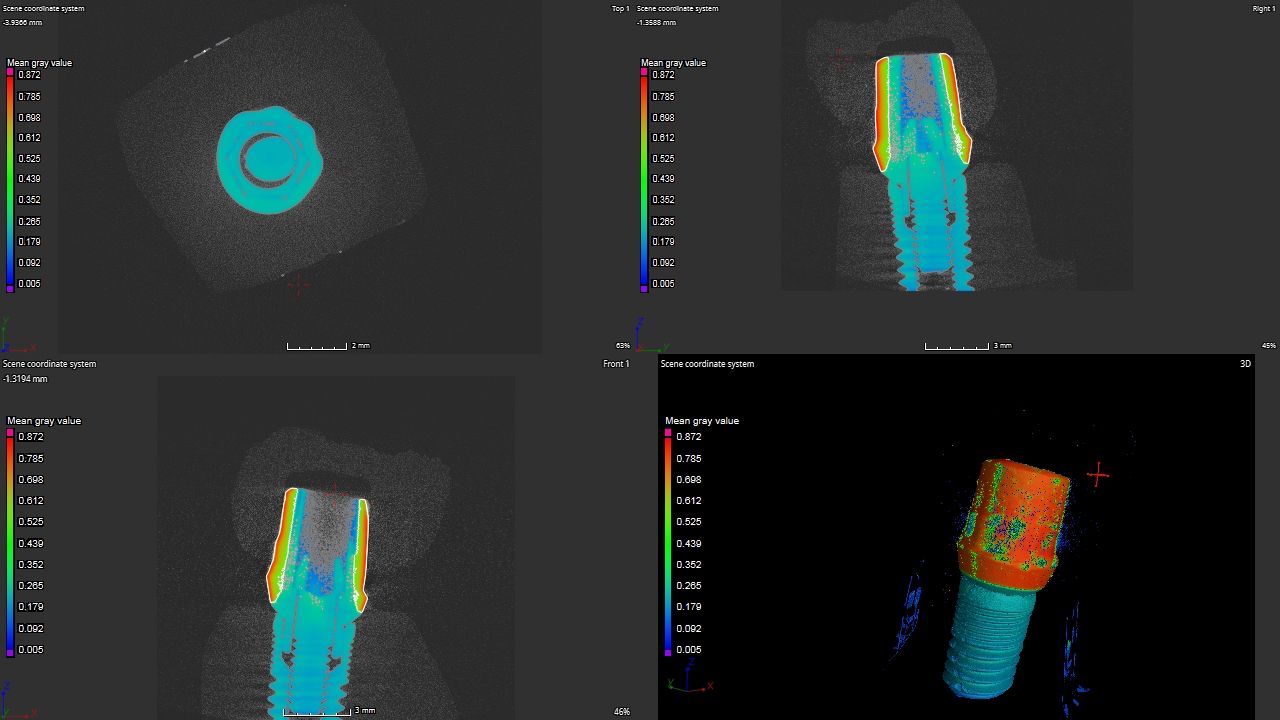

Supplement: Supplementary file 1 — Supplementary Material 1. [file 12903_2026_8150_MOESM1_ESM.zip › 6.jpeg]

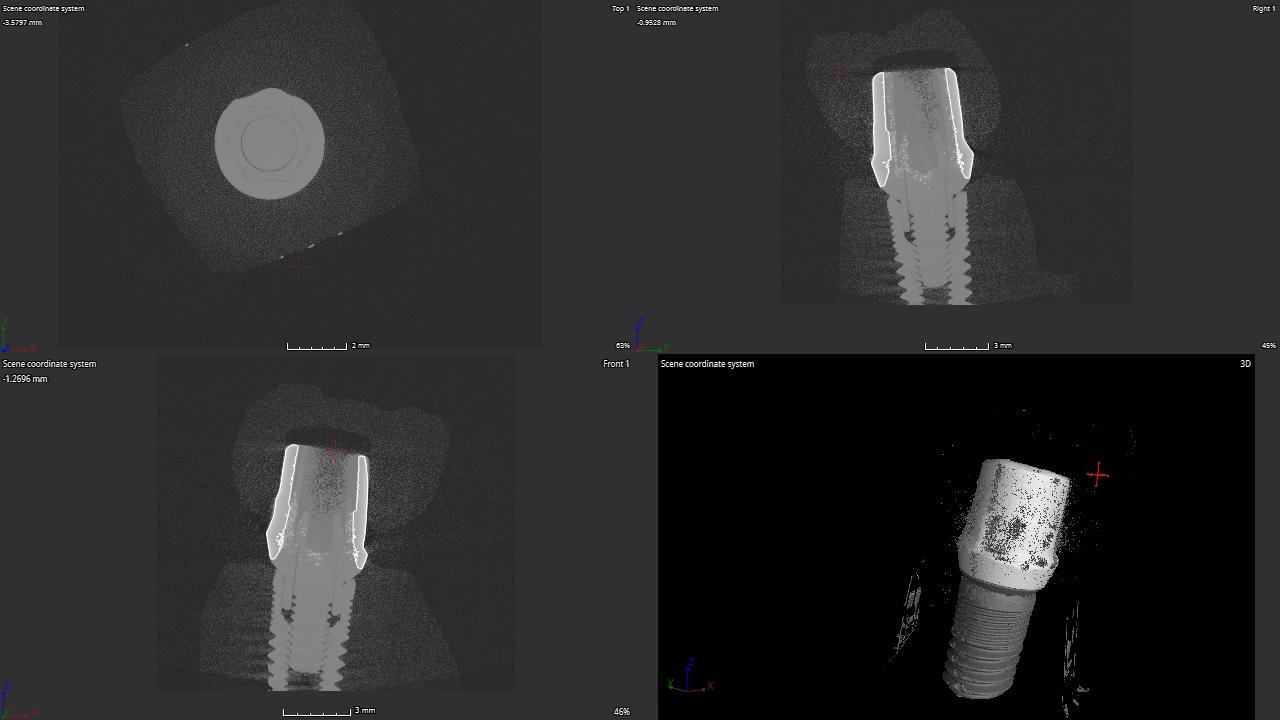

Supplement: Supplementary file 1 — Supplementary Material 1. [file 12903_2026_8150_MOESM1_ESM.zip › 7.jpeg]

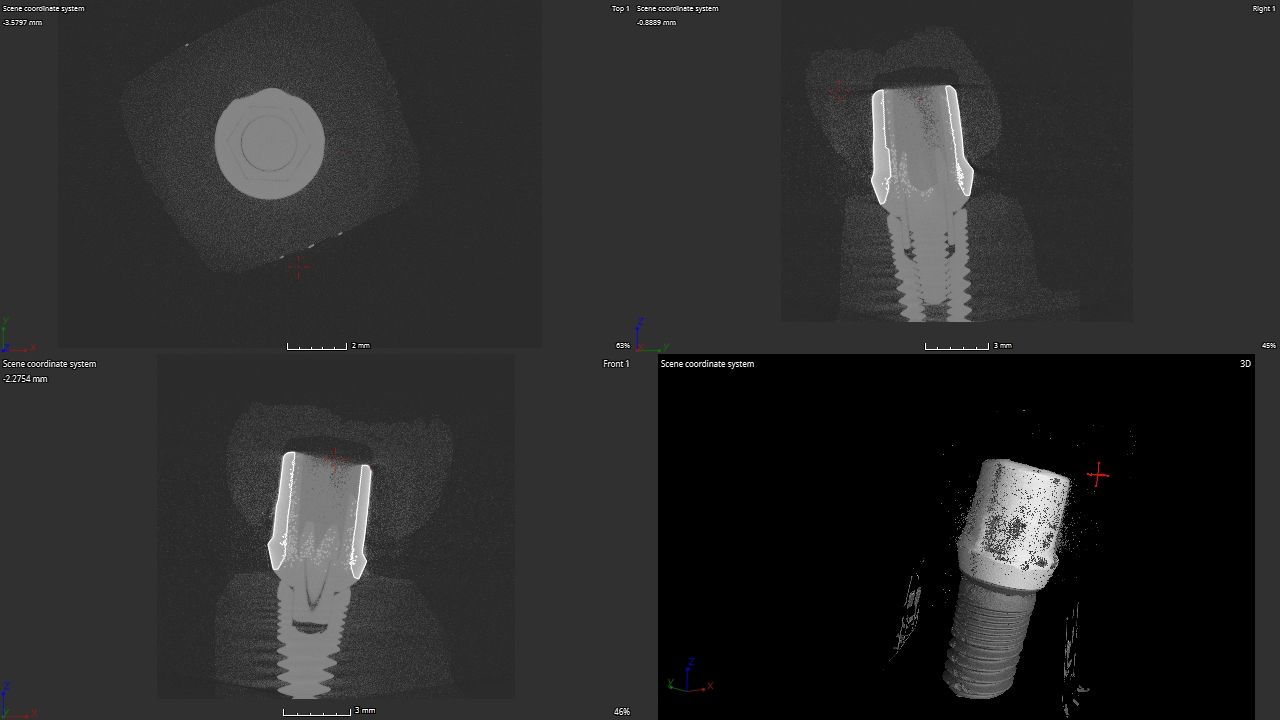

Supplement: Supplementary file 1 — Supplementary Material 1. [file 12903_2026_8150_MOESM1_ESM.zip › 8.jpeg]

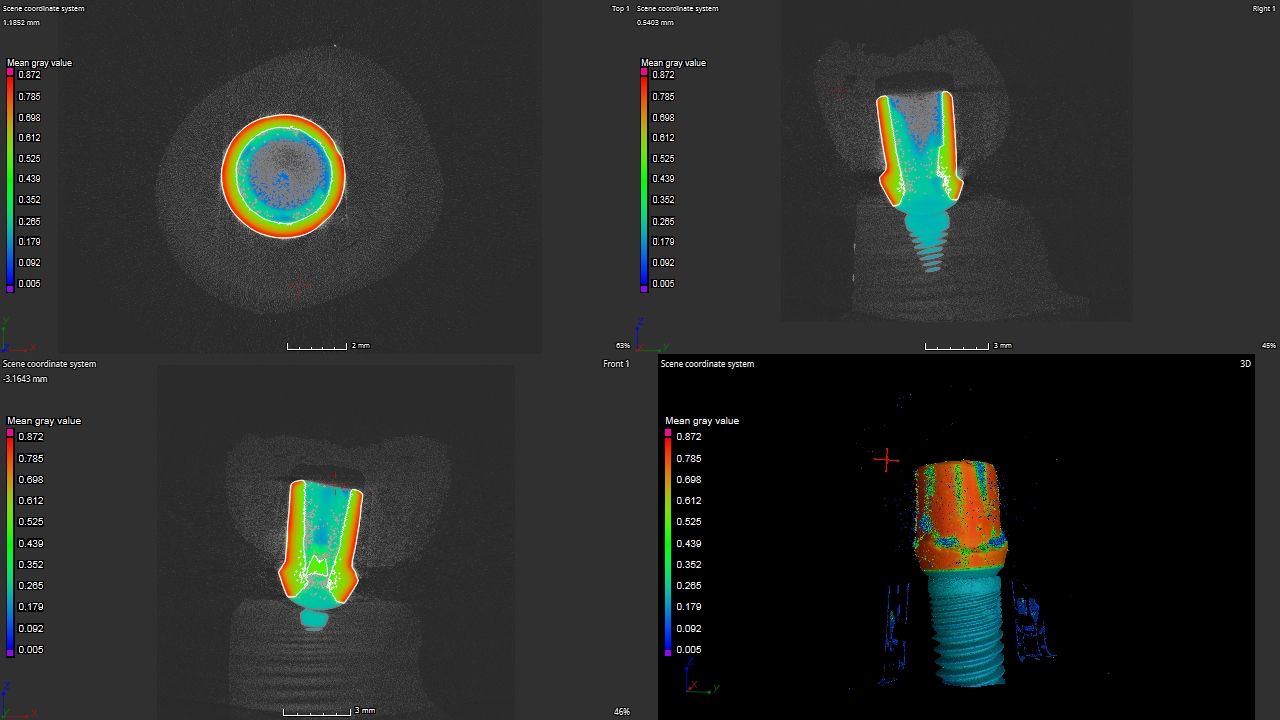

Supplement: Supplementary file 1 — Supplementary Material 1. [file 12903_2026_8150_MOESM1_ESM.zip › 11.jpeg]

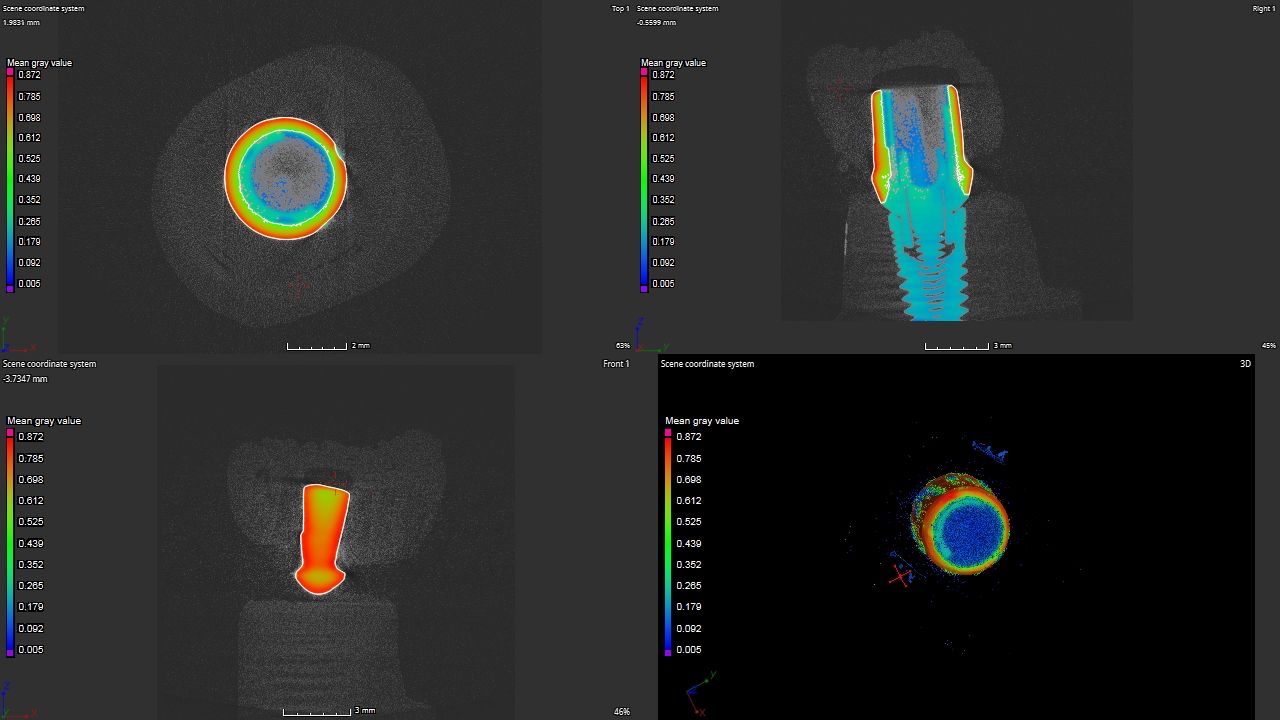

Supplement: Supplementary file 1 — Supplementary Material 1. [file 12903_2026_8150_MOESM1_ESM.zip › 12.jpeg]
